# DCAF1 interacts with PARD3 to promote hepatocellular carcinoma progression and metastasis by activating the Akt signaling pathway

**DOI:** 10.1186/s13046-024-03055-2

**Published:** 2024-05-06

**Authors:** Jinyao Zhang, Yuze Shi, Ke Ding, Weiwei Yu, Jianbo He, Beicheng Sun

**Affiliations:** 1 Department of Hepatobiliary Surgery, Nanjing Drum Tower Hospital, Chinese Academy of Medical Sciences & Peking Union Medical College, Peking Union Medical College Graduate School, Nanjing, Jiangsu Province 210008 China; 2 Department of Hepatobiliary Surgery, The Affiliated Drum Tower Hospital of Nanjing University Medical School, Nanjing, Jiangsu Province 210008 China; 3 Department of Hepatobiliary Surgery, Nanjing Drum Tower Hospital Clinical College of Nanjing Medical University, Nanjing, Jiangsu Province 210008 China; 4 Department of Thoracic and Cardiovascular Surgery, The Affiliated Drum Tower Hospital of Nanjing University Medical School, Nanjing, Jiangsu Province 210008 China; 5 Department of Hepatobiliary Surgery, The First Affiliated Hospital of Anhui Medical University, Hefei, Anhui Province 230022 China

**Keywords:** Hepatocellular carcinoma, DCAF1, PARD3, Akt

## Abstract

**Background:**

Hepatocellular carcinoma (HCC) is a fatal malignancy with poor prognosis due to lack of effective clinical interference. DCAF1 plays a vital role in regulating cell growth and proliferation, and is involved in the progression of various malignancies. However, the function of DCAF1 in HCC development and the underlying mechanism are still unknown. This study aimed to explore the effect of DCAF1 in HCC and the corresponding molecular mechanism.

**Methods:**

Quantitative real-time PCR, Western blot and immunostaining were used to determine DCAF1 expression in tumor tissues and cell lines. Subsequently, in vitro and in vivo experiments were conducted to explore the function of DCAF1 in tumor growth and metastasis in HCC. Coimmunoprecipitation, mass spectrometry and RNA sequencing were performed to identify the underlying molecular mechanisms.

**Results:**

In this study, we found that DCAF1 was observably upregulated and associated with poor prognosis in HCC. Knockdown of DCAF1 inhibited tumor proliferation and metastasis and promoted tumor apoptosis, whereas overexpressing DCAF1 yielded opposite effects. Mechanistically, DCAF1 could activate the Akt signaling pathway by binding to PARD3 and enhancing its expression. We also found that the combined application of DCAF1 knockdown and Akt inhibitor could significantly suppress subcutaneous xenograft tumor growth.

**Conclusions:**

Our study illustrates that DCAF1 plays a crucial role in HCC development and the DCAF1/PARD3/Akt axis presents a potentially effective therapeutic strategy for HCC.

**Graphical Abstract:**

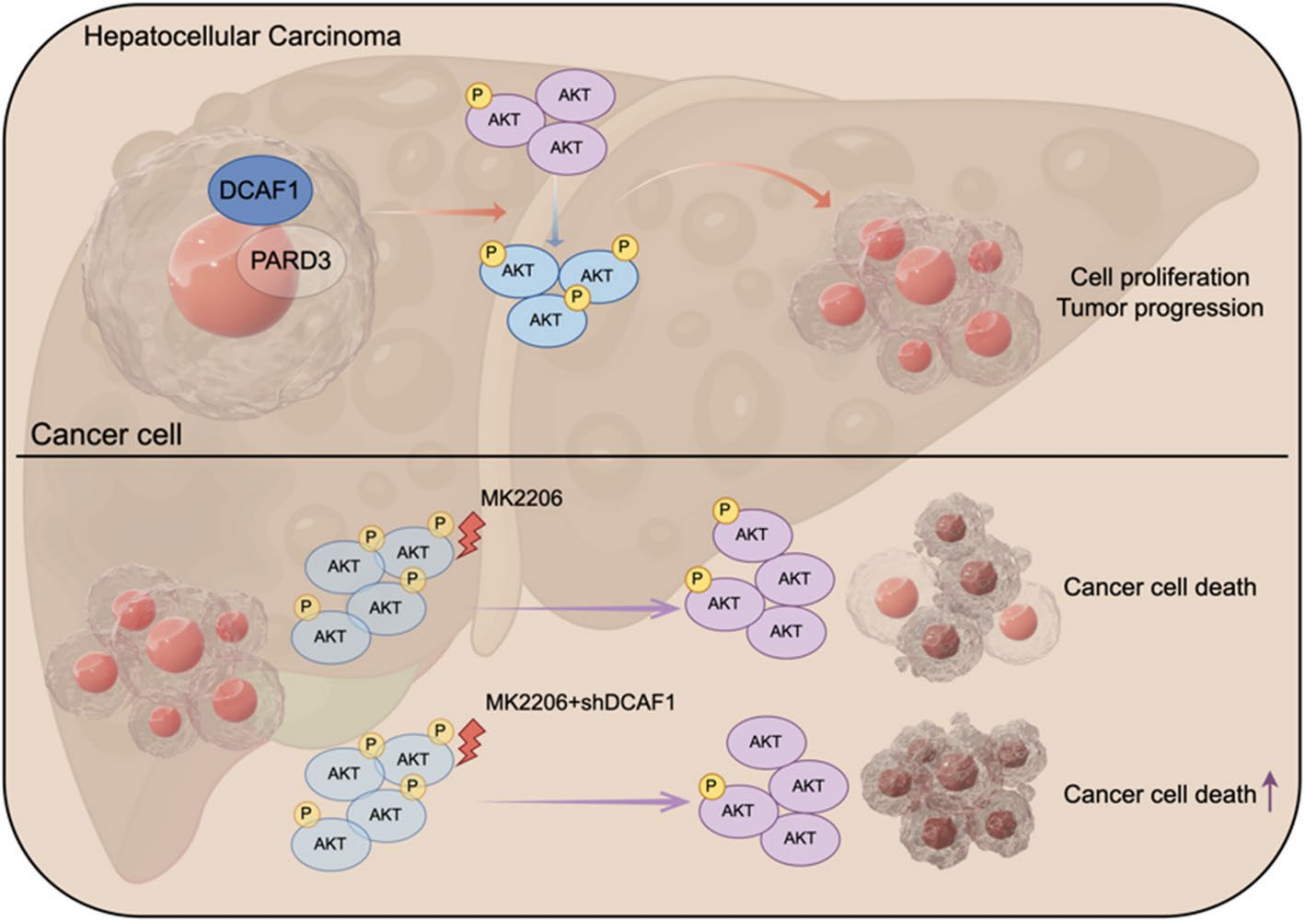

**Supplementary Information:**

The online version contains supplementary material available at 10.1186/s13046-024-03055-2.

## Background

Liver cancer is one of the most fatal malignancies, with a 5-year survival rate of only 21% [1]. Hepatocellular carcinoma (HCC) is the most common type of liver cancer, accounting for approximately 90% of the total [2]. Despite the constantly updated clinical treatment, the overall survival of HCC patients has not been obviously ameliorated. It is necessary to seek novel molecular mechanisms to facilitate precision treatment for HCC.

The DDB1-CUL4-associated factors (DCAFs) are substrate receptors for CRL4, an E3 ubiquitin ligase, and contain more than 100 members. DDB1-CUL4-associated factor 1 (DCAF1), originally discovered as HIV-1 Vpr Binding Protein (VprBP) [3], belongs to DCAFs and participates in various physiological and pathological processes [4], including cell growth and proliferation [5], virus replication [6] and transcriptional repression [7]. It has been reported that DCAFs are involved in various malignant tumors [4]. For instance, DCAF3 could promote metastasis of breast cancer [8], and DCAF13 could enhance cell proliferation and growth in head and neck squamous cell carcinoma [9]. Moreover, DCAF1 is associated with several types of malignancies, including ovarian cancer, colon cancer and prostate cancer [10–12]. However, the functional role that DCAF1 plays in HCC is still unknown.

Par-3 family cell polarity regulator (PARD3) is a member of the Par polarity complex, which governs cell polarity [13]. PARD3 is reported to be dysregulated and to participate in the progression of several types of malignancies, high expression of PARD3 predicts poor prognosis in HCC [14]. And a recent study has found that PARD3 drives tumorigenesis through activating Sonic Hedgehog signalling in tumour-initiating cells in liver cancer [15]. Meanwhile, in CRC, PARD3 was found interacted with Trip6, promoted EMT and activated Akt signaling [16].

The study indicated that DCAF1 was markedly upregulated in HCC and that downregulated DCAF1 inhibited the proliferation and migration of HCC cells. Furthermore, our experiments elucidated that PARD3 bound to and was upregulated by DCAF1 in HCC, and knockdown of PARD3 could partially counteract the promotive effect of DCAF1 on HCC. In addition, downregulated DCAF1 suppressed the Akt signaling pathway in HCC, and DCAF1 knockdown combined with an Akt inhibitor showed synergistic suppression of HCC progression. Our study illustrates that DCAF1 plays a crucial role in HCC development and the DCAF1/PARD3/Akt axis presents a potentially effective therapeutic strategy for HCC.

## Materials and methods

### Clinical specimens

The tumor and paired adjacent peritumor tissues were obtained from patients undergoing liver resection at the Department of Hepatobiliary Surgery, Nanjing Drum Tower Hospital. Normal liver tissues were collected from patients who received hepatectomy owing to hepatic hemangioma. With the approval of the ethics committee of our institute, all patients signed informed consent for the use of specimens before surgery. Fresh tissues were collected and placed in liquid nitrogen within ten minutes of being excised. All studies were conducted in accordance with national policies and the Helsinki declaration.

### Database and analysis

The GSE63898 dataset was involved to analyze the DCAF1 mRNA expression in tumor and non-tumor tissues. In this dataset, tumor tissues were collected from 228 HCC patients, and non-tumor tissues were from 168 patients with liver cirrhosis. Overall survival and Clinic pathologic correlations were analyzed using *survival* and *survminer* in R software or SPSS based on the TCGA-LIHC dataset which contains 365 HCC patients’ information. In details, the optimal Cut-off value set by *survminer* is 10.78546. Value of (VprBp)DCAF1 over than 10.78546 referred as “High level”, less than 10.78546 referred as “Low level”.

### Cell lines and cell culture

Human hepatic carcinoma cell lines (Hep3B, Huh7, SMMC-7721, HepG2, MHCC-97H and LM3) and immortalized hepatocyte HepRG cells were purchased from the Shanghai Institute for Biological Science (China). All cells were cultured in Dulbecco’s modified Eagle’s medium (DMEM) with 10% FBS, 100 U/mL penicillin, and 100 mg/mL streptomycin and incubated in a humid environment at 37 °C with 5% CO2. The Akt activator SC79 and Akt inhibitor MK2206 were purchased from Beyotime (China). The working concentration of SC79 and MK2206 was 5 μM.

### Cell transfection

Lentiviruses stably expressing DCAF1^+^/PARD3^+^/DCAF1-shRNA/PARD3-shRNA were transfected into HCC cells cultured in six-well plates and treated with polybrene (10 µg/ml) for 24 h. Details of the shRNA sequence were provided in supplementary Table 1. DCAF1 deletion mutants were transiently transfected into HCC cells cultured in 10 cm dishes with using Lipo8000™ (Beyotime, #C0533). The segments design was shown in Supplementary Table 2.

### Cell viability assay

The viability of cells was detected using a CCK8 kit (Vazyme, #A311) and EdU Cell Proliferation Kit with Alexa Fluor 555 or 488 (Beyotime, #C0071&C0075). For the CCK8 proliferation experiment, cells were seeded in 96-well plates at a density of 1,000 cells per well and cultured overnight. The cells were washed twice with sterile PBS; then, 10 µl of CCK8 reagent and 90 µl of serum-free medium were added to each well. Then, the cells were incubated for 1 h at 37°C. The absorbance at 450 nm of the sample was measured using a microplate reader. For the EdU assay, cells were seeded into 24-well plates (3 × 10^5^ cells/well) and treated in accordance with the manufacturer’s instructions. Finally, images were taken with fluorescence microscopy.

### Transwell migration and invasion assays

To examine the cell migration and invasion ability, transwell assays with or without Matrigel were performed. A total of 2× 10^4^ cells were placed in the upper chamber, and the substratum chamber contained 600 μl of DMEM with 10% FBS. The cells were incubated for 48 h, and then the chamber was removed and rinsed with PBS. The chamber was then treated with 4% formaldehyde for 15 min and stained with crystal violet. The cells that remained on top of the membrane were scraped off gently with a cotton swab. Then, the cells were viewed and captured under a light microscope.

### Colony formation

A total of 1× 10^3^ cells were seeded into each well of a 6-well plate and cultured for two weeks. The medium was replaced every four days. The cells were fixed with neutral polyformaldehyde for 30 min at room temperature and then stained with 0.1% crystal violet. Then, the colonies that contained more than 50 cells were counted.

### Wound-healing assay

Approximately 6 × 10^4^ cells were added to each well of an *Ibidi Culture-Insert 2 Well* in a 24-well plate. After the cells were attached, a sterile tweezer was used to remove the Culture-Insert. DMEM containing 1% FBS was added to the dish after washing with PBS twice. Wound closure was observed and photographed by an Olympus optical microscope and MShot Image Analysis System.

### RNA isolation and quantitative real-time PCR (qRT‒PCR)

RNAs from liver tissues and cells were isolated using TRIzol reagent (Invitrogen, #15,596,018) with reference to the manufacturer’s protocol. The RNAs were reverse transcribed into cDNA by using HiScript III RT SuperMix (Vazyme, #R323-01), followed by qRT‒PCR. It was conducted as stated in the manual of the ChamQ Universal SYBR qPCR Master Mix (Vazyme, #Q711-02) with an Applied Biosystems 7300 Detection System (Applied Biosystems®, CA). The β-actin levels were used as internal controls to normalize the data obtained. The sequences of specific primers used in this study were listed in Supplementary Table 3.

### Western blot

Cells and liver tissues were treated with ice-cold RIPA buffer containing newly added PMSF solution (Beyotime, #ST507) and phosphatase inhibitors (KeyGEN, #KGP602) to extract proteins. BCA assay was then conducted to determine the concentrations. Subsequently, the protein samples were subjected to SDS‒PAGE for separation, followed by transfer onto a PVDF membrane (Bio-Rad). Immunoblotting was performed by blocking the membrane with 5% BSA in TBST buffer, incubating with specific primary antibodies, and then with appropriate secondary antibodies. Protein visualization was achieved using an enhanced chemiluminescence detection kit (Vazyme, #E412-01). The pertinent details regarding the antibodies used in Western blot can be found in Supplementary Table 4.

### Coimmunoprecipitation (coIP)

Related cultured cells were washed with phosphate-buffered saline and ruptured using lysis buffer (Beyotime, #P0013) containing newly added PMSF solution (Beyotime, #ST507) and phosphatase inhibitors (KeyGEN, #KGP602). The lysates were then incubated on ice for 30 min and centrifuged at 12,000 rpm for 15 min. The resulting supernatant was collected, and the protein concentration was measured using the BCA Assay Kit (Beyotime, #P0011). For subsequent analysis, proteins were either directly used for Western blot as input or subjected to immunoprecipitation. Specifically, the proteins were incubated with primary antibodies and BeyoMag™ Protein A + G Magnetic Beads (Beyotime, #P2108) at 4°C overnight. The beads were then collected using a magnetic stand, washed three times with Wash Buffer, and resuspended in loading buffer. In the end, the beads were heated at 95°C for 5 min, and the supernatant was subsequently subjected to SDS‒PAGE. The relevant information of the antibodies used in coIP assay is available in Supplementary Table 5.

### Mass spectrometry

Proteins isolated from HepG2 cells overexpressing DCAF1 were subjected to immunoprecipitation using DCAF1 primary antibody. Subsequently, the immunoprecipitated proteins were separated by SDS‒PAGE and stained with Coomassie brilliant blue. After proteins were enzymatically digested into peptides, chromatographic separation was performed using the nanoflow Easy nLC 1200 chromatography system (Thermo Scientific). The separated peptides were then subjected to data-dependent acquisition (DDA) mass spectrometry analysis using a Q Exactive mass spectrometer (Thermo Scientific). The mass spectrometry database retrieval software was MaxQuant 2.0.1. The following protein dataset was employed: Database: UniProt-Reference Proteome-*Homo sapiens* (Human) [9606]-20607–20221014.fasta, sourced from the website https://www.uniprot.org/proteomes/UP000005640.

### Immunofluorescence (IF) and immunohistochemistry (IHC) assays

For the immunofluorescence assay, 4-μm thick fresh frozen sections were fixed with 4% paraformaldehyde for 30 min. After permeabilization and blocking, the sections were incubated with primary antibody at 4°C overnight. The sections were then washed with PBS three times and stained with Alexa Fluor 488-conjugated anti-rabbit secondary antibody for 2 h at room temperature. Nuclei were stained with DAPI for 20 min. The images were taken by using an inverted confocal fluorescence microscope (Leica Microsystems CMS GmbH, TCS SP8) and software (Leica Application Suite X, 3.6.20104.0).

For the immunohistochemistry experiment, Paraffin sections were dewaxed and rehydrated. followed by antigen retrieval. Then sections were incubated with primary antibody overnight at 4°C. Then, biotin-conjugated secondary antibody was added. Detection was finally conducted using 3,3'-diaminobenzidine and hematoxylin. The relevant information of the antibodies used in IF and IHC assays can be found in Supplementarys Table 6 and 7.

### Evaluation of IHC staining

The protein expressions in immunohistochemically stained tissues were semi-quantitatively assessed using the Histological Score (H-Score) system [17]. The H-Score was calculated by multiplying the percentage of positive area with the intensity of staining. Intensity was categorized as weak, moderate and strong, denoted by numeric values from 1 to 3, respectively (weak: 1, moderate: 2, strong: 3). The H-Score was determined using the formula: H-score = 1 × (% of faintly stained tumor area) + 2 × (% of moderately stained tumor area) + 3 × (% of strongly stained tumor area). Consequently, the H-score ranged from 0 to 300.

### Flow cytometry analysis

An Annexin V-FITC/PI Apoptosis Detection Kit (Vazyme, #A211-01) was used to evaluate apoptosis. The cells were seeded into a six-well plate and cultured in complete medium at 37°C until reaching 80% confluence and then stimulated with 0.05 mM H_2_O_2_ for 2 h to induce apoptosis. Following digestion with non-EDTA trypsin, cells were collected and stained with PI and FITC for 10 min at room temperature in the dark. A Cell Cycle and Apoptosis Analysis Kit (Beyotime, #C1052) was applied to test the cell cycle. The cells were seeded into a six-well plate and cultured in complete medium at 37°C overnight. After digestion with non-EDTA trypsin, the cells were collected, fixed with 70% ethanol at 4°C overnight and subsequently stained with PI for 30 min at 37°C in the dark. Finally, flow cytometry analysis was carried out on DxP Athena flow cytometer (Cytek Biosciences, USA) equipped with FlowJo Software 6.0 (BD Biosciences, New York, NY, USA).

### RNA sequencing and differentially expressed genes analysis

Total RNA from cultured cells was extracted with TRIzol reagent (Invitrogen, #15,596,018). After evaluating RNA purity and quantification and assessing RNA integrity, the libraries were constructed and then sequenced on an Illumina NovaSeq 6000 platform. Differential expression analysis was performed using the R package DESeq2. Q value < 0.05 and fold change > 2 or fold change < 0.5 was set as the thresholds for significantly differentially expressed genes (DEGs). KEGG pathway enrichment analysis of DEGs in both Hep3B and SMMC-7721 cells was performed using R software.

### Tumorigenesis assays in nude mice

Six-week-old male BALB/c nude mice were housed in groups of five mice per cage in a specific pathogen-free room with controlled temperature and lighting conditions at 25°C ± 1°C and a 12-h light–dark cycle. Prior to the experiment, a one-week acclimatization period was allowed for all mice. To examine whether DCAF1 influences HCC growth in vivo, the experimental group and control group of cells were injected into the left and right inguinal regions of mice (3.0 × 10^6^ cells per side) to establish subcutaneous tumor models. For DCAF1 knockdown and Akt inhibitor combined application experiments, 3.0 × 10^6^ Hep3B cells transfected with DCAF1-shRNA or control vector were subcutaneously inoculated into the left or right groin of mice, respectively. One week later, the mice were randomly allocated into vehicle or MK2206 groups (*n* = 6 per group). MK2206 was orally administered to mice three times a week at a dose of 120 mg/kg. Tumor volumes were measured every 4 days. At the end of the experiment, the mice were sacrificed, and the tumors were surgically removed and weighed. Then, the tumors were subjected to IHC staining and Western blot.

### Statistical analysis

GraphPad Prism 9 (GraphPad Inc., La Jolla, CA, USA) and IBM SPSS Statistics 26 were used for statistical analyses. All experiments were independently performed at least 3 times, and the results are expressed as the mean ± standard deviation (S.D.). The differences between two or more groups were analyzed by unpaired Student’s t test or one-way ANOVA followed by Dunnett’s multiple comparisons test. The correlation between the DCAF1 expression level and clinicopathologic features of HCC patients was analyzed by the χ2 test. *P* value < 0.05 was considered statistically significant for all tests.

## Results

### DCAF1 is overexpressed in HCC tissues and related to poor prognosis

**Fig. 1:**
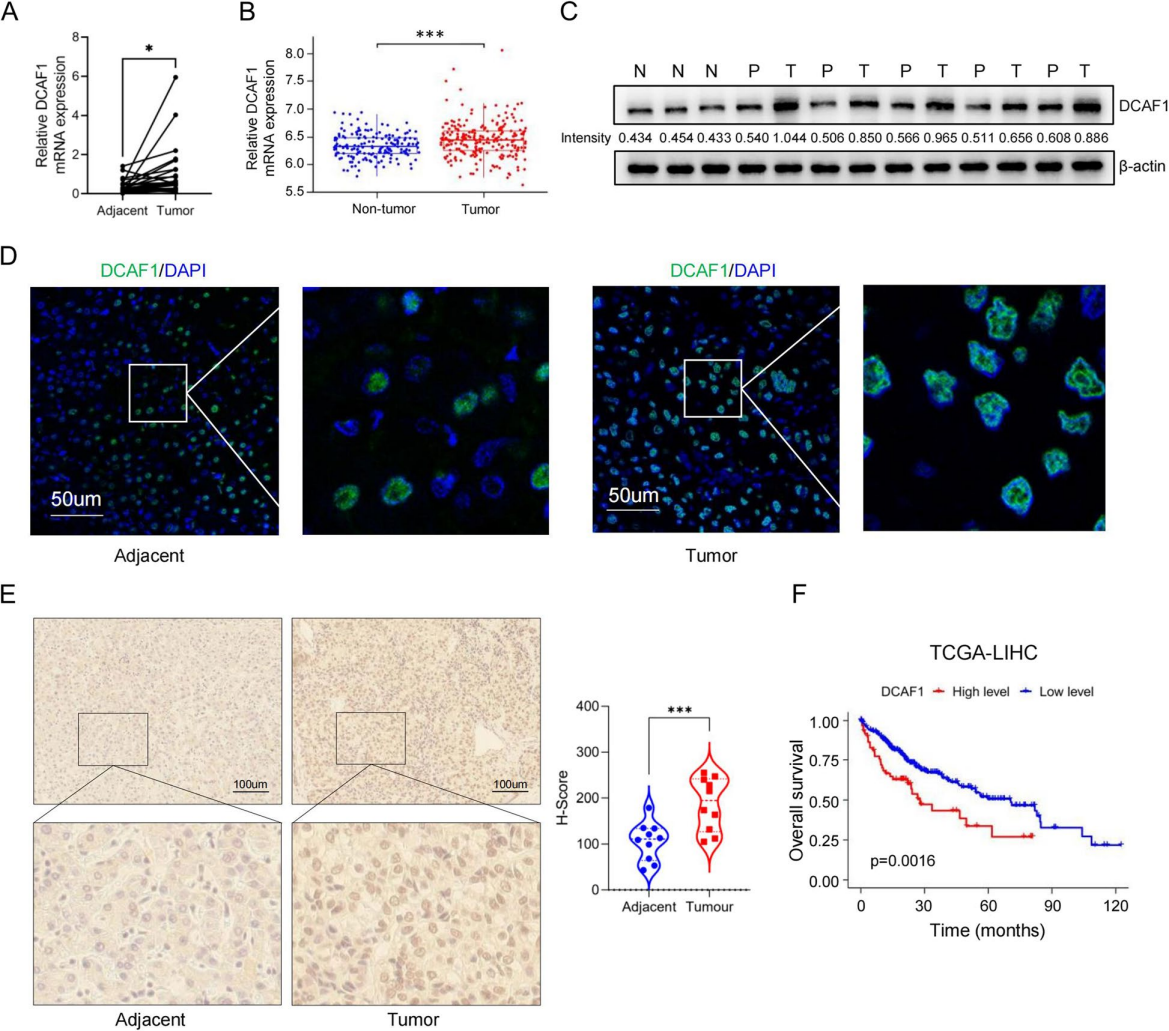
DCAF1 is upregulated in HCC tissues and correlated with poor prognosis. **A** Related mRNA expression of DCAF1 in HCC and adjacent peritumor tissues tested by qRT‒PCR. **B** Related mRNA expression of DCAF1 in the public dataset GSE63898. **C** Related protein expression of DCAF1 in tumor (T), adjacent peritumor (P) and normal (N) tissues tested by Western blot. **D**-**E** DCAF1 expression in HCC tissues and adjacent peritumor tissues was tested by IF **D** and IHC **E** assays (Bar = 50μm(D), Bar = 100μm(E)).** F** Kaplan–Meier analysis of the overall survival of patients from the DCAF1 high and low expression groups in the TCGA-LIHC dataset. * *p* < 0.05, *** *p* < 0.001

We initially examined DCAF1 mRNA expression in 26 pairs of tumor and adjacent peritumor tissues from HCC patients using qRT-PCR. The mRNA expression level of DCAF1 was significantly elevated in HCC tissues compared to adjacent peritumor tissues (Fig. 1A). Similarly, in the public dataset GSE63898, there was a significant increase in DCAF1 mRNA level in tumor tissues compared to non-tumor tissues (Fig. 1B). Subsequently, Western blot analysis was conducted to investigate the protein expression of DCAF1. The DCAF1 protein level (examined in 5 pairs) was increased in tumors compared to adjacent peritumor tissues and was further elevated compared to normal tissues (Fig. 1C). Similarly, both immunofluorescence (IF) (Fig. 1D) and immunohistochemistry (IHC) results (examined in 10 pairs) (Fig. 1E) consistently confirmed a significant upregulation of DCAF1 at the protein level in HCC tissues compared to adjacent peritumor tissues. Clinically, HCC patients with high DCAF1 expression exhibited shorter overall survival in the TCGA-LIHC cohort (Fig. 1F). Furthermore, the DCAF1 expression level was correlated with AJCC stage and histologic grade in the TCGA-LIHC cohort (Table 1).

**Table 1:** Correlation between DCAF1 expression level and clinicopathological features of HCC patients

Clinical characteristics	Total	DCAF1 expression, n (%)		*X*^*2*^	*P*
		**Low**	**High**		
Gender				0.319	0.654
Male	246	203 (82.5)	43 (17.5)		
Female	119	101 (84.9)	18 (15.1)		
Age (years)				0.314	0.567
≤ 65	227	191 (84.1)	36 (15.9)		
> 65	138	113 (81.9)	25 (18.1)		
AFP (ng/ml)				0.077	0.859
≤ 200	201	166 (82.6)	35 (17.4)		
> 200	75	63 (84.0)	12 (16.0)		
NA	89	75 (84.3)	14 (15.7)		
T classification				5.360	0.147
T1	183	159 (86.9)	24 (13.1)		
T2	91	76 (83.5)	15 (16.5)		
T3	78	59 (75.6)	19 (24.4)		
T4	13	10 (76.9)	3 (23.1)		
AJCC stage				**4.902**	**0.031**
Stage I + II	255	219 (85.9)	36 (14.1)		
Stage III + IV	86	65 (75.6)	21 (24.4)		
NA	24	20 (83.3)	4 (16.7)		
Histologic grade				**11.868**	**0.003**
G1 + G2	230	203 (88.3)	27 (11.7)		
G3 + G4	130	98 (75.4)	32 (24.6)		
NA	5	3 (60.0)	2 (40.0)		
Vascular invasion				3.137	0.208
Absent	205	177 (86.3)	28 (13.7)		
Present	106	84 (79.2)	22 (20.8)		
NA	54	43 (79.6)	11 (20.4)		

Data were analyzed by chi-squared test. *P* values in bold indicate statistical significance

### DCAF1 facilitates HCC proliferation in vitro and in vivo

**Fig. 2:**
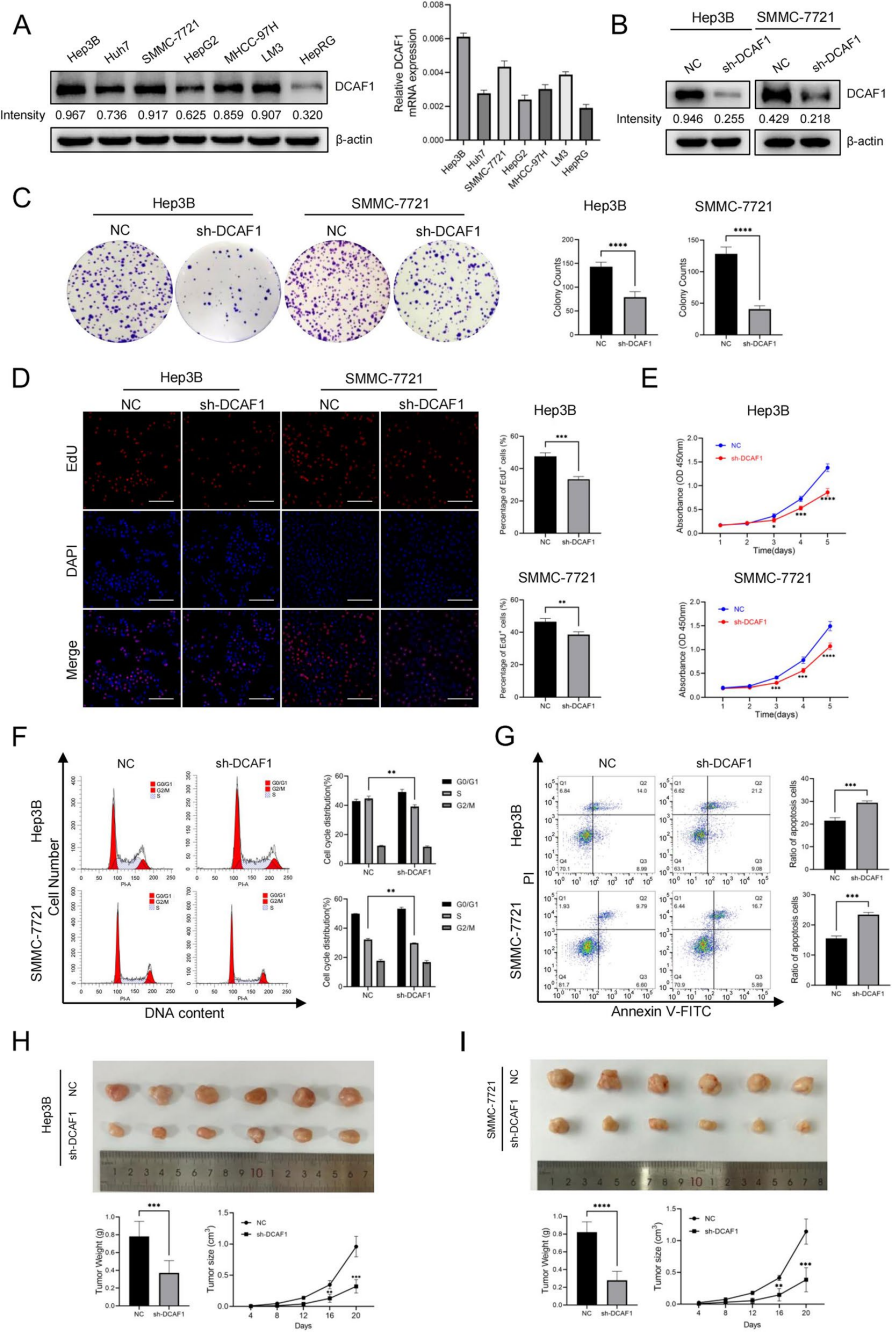
DCAF1 promotes HCC proliferation in vitro and in vivo. **A** Expression of DCAF1 in HCC cell lines and HepRG cells detected by Western blot and qRT‒PCR. **B** The efficacy of DCAF1 knockdown in Hep3B and SMMC-7721 cells verified by Western blot. **C** The colony formation results of Hep3B and SMMC-7721 cells after knockdown of DCAF1. **D** EdU assays results of Hep3B and SMMC-7721 cells after DCAF1 knockdown (Bar = 100μm). **E** CCK8 assays of Hep3B and SMMC-7721 cells after knockdown of DCAF1. **F** Cell cycle analysis of Hep3B and SMMC-7721 cells was conducted by flow cytometry. **G** The cell apoptosis ratio of Hep3B and SMMC-7721 cells was detected by flow cytometry using the Annexin V-FITC/PI staining kit. **H** Hep3B cells with DCAF1 knockdown were transplanted on nude mice, and tumor volumes and tumor weights were recorded. **I** SMMC-7721 cells with DCAF1 knockdown were transplanted on nude mice, and tumor volumes and tumor weights were recorded. Data are shown as the mean ± SD. * *p* < 0.05, ** *p* < 0.01, *** *p* < 0.001, **** *p* < 0.0001

Both Western blot and qRT‒PCR analyses revealed a marked increase in DCAF1 expression in HCC cell lines compared to HepRG cells (Fig. 2A). Notably, Hep3B and SMMC-7721 cells exhibited the highest, while HepG2 cells displayed the lowest DCAF1 expression levels (Fig. 2A). To investigate the functional role of DCAF1 in HCC, we employed DCAF1 knockdown in Hep3B and SMMC-7721 cells (Fig. 1B), and DCAF1 overexpression in HepG2 cells (Figure S1B). Subsequent in vitro assays, including colony formation, EdU and CCK8 assays, demonstrated that cell proliferation was inhibited upon DCAF1 knockdown, while it was enhanced with DCAF1 overexpression (Fig. 2C-E, S1C-E). Moreover, flow cytometry analysis revealed that DCAF1 knockdown resulted in an augmented proportion of cells in the G0/G1 phase, along with a decline in the S phase (Fig. 2F). Conversely, overexpression of DCAF1 led to a decreased proportion of cells in the G0/G1 phase, accompanied by an increase in the S phase (Figure S1F). These findings provide compelling evidence that the reduction of DCAF1 induces cell cycle arrest at the G0/G1 phase. Additionally, DCAF1 knockdown increased the apoptotic rate (Fig. 2G), while overexpressing DCAF1 yielded the opposite effect (Figure S1G). Collectively, these results suggest that DCAF1 promotes HCC cell proliferation in vitro.

To assess the impact of DCAF1 on HCC proliferation in vivo, Hep3B cells stably transfected with DCAF1-shRNA and HepG2 cells stably overexpressing DCAF1 were subcutaneously injected into BALB/c nude mice. As depicted in Fig. 2H and I, the tumors in the DCAF1 knockdown group exhibited a significant reduction in size and weight compared to the control group. In contrast, tumors in the DCAF1 overexpression group displayed increased volume and weight compared to the pCDH group (Figure S1H). Furthermore, expression of Ki67 and DCAF1 in the tumor tissues was detected by IHC staining, and H-Score was then calculated. The results showed that the expression of Ki67 significantly reduced after DCAF1 knockdown and remarkably increased with DCAF1 overexpression (Figure S1I-K). These results collectively affirm that DCAF1 facilitates tumor growth in vivo.

### DCAF1 enhances HCC metastasis and promotes epithelial-mesenchymal transition

**Fig. 3:**
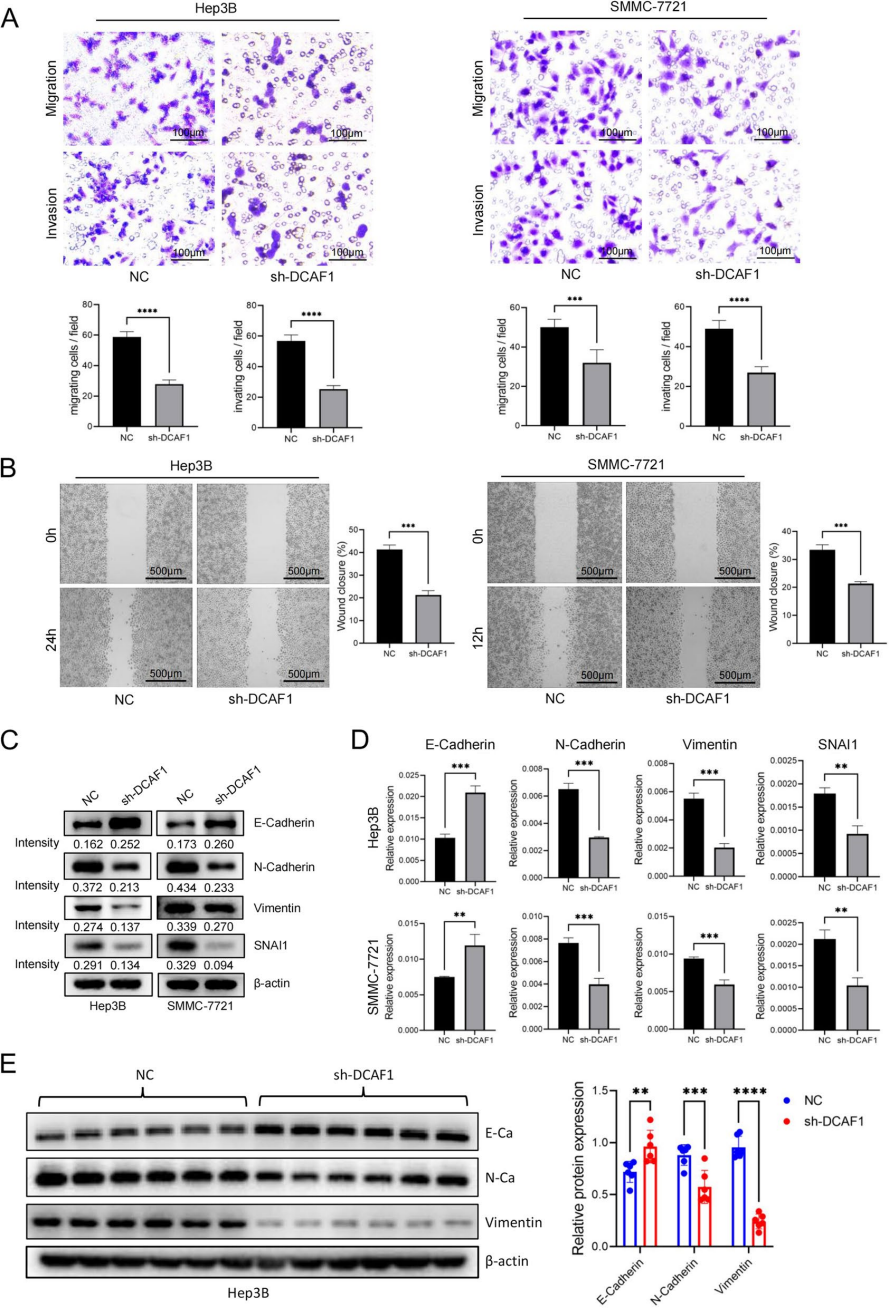
DCAF1 regulates cell migration and invasion in HCC cell lines and activates EMT.** A** Representative image of migrated and invaded Hep3B and SMMC-7721 cells after DCAF1 knockdown (Bar = 100μm). **B** Wound healing assays were performed to assess the effect of DCAF1 knockdown on cell motility in HCC cells (Bar = 500μm). **C** The protein expression levels of EMT markers in Hep3B and SMMC-7721 cells after knockdown of DCAF1 were detected by Western blot. **D** The mRNA expression levels of EMT markers in Hep3B and SMMC-7721 cells after knockdown of DCAF1 were detected by qRT‒PCR. Data are shown as the mean ± SD. **E** The expression of EMT marker proteins in xenografts was assessed by Western blot. ** *p* < 0.01, *** *p* < 0.001, **** *p* < 0.0001

The results of transwell and wound-healing experiments showed that knockdown of DCAF1 suppressed the migration and invasion of Hep3B and SMMC-7721 cells (Fig. 3A-B), and overexpressing DCAF1 in HepG2 cells enhanced cell migration and invasion ability (Figure S2A, B). Epithelial-mesenchymal transition (EMT) is a process that can impart metastatic properties to malignant cells by enhancing mobility, invasiveness, and resistance to apoptotic stimuli [18]. Prior investigations have indicated that EMT plays a vital role in HCC progression [19]. To explore whether DCAF1 regulates EMT in HCC, protein and mRNA expression of EMT markers was detected by Western blot (Fig. 3C, S2C) and qRT‒PCR (Fig. 3D, S2D). The results showed that suppressing DCAF1 led to a reduction in N-cadherin, vimentin and SNAI1 expression levels, as well as an increase in E-cadherin expression level. Conversely, overexpression of DCAF1 resulted in elevated expression levels of N-cadherin, vimentin and SNAI1, along with a concomitant decrease in the expression of E-cadherin.

Additionally, the expression levels of EMT marker protein in mice xenografts were assessed by Western blot (Fig. 3E, S2E). A significant reduction in mesenchymal marker proteins and increase in epithelial marker protein could be seen after DCAF1 knockdown. Conversely, when DCAF1 was overexpressed, the expression levels of mesenchymal marker proteins increased, while the expression of E-cadherin decreased. The above findings collectively demonstrate that DCAF1 enhances HCC metastasis and promotes EMT.

### DCAF1 associates with and upregulates PARD3 to promote tumor growth

**Fig. 4:**
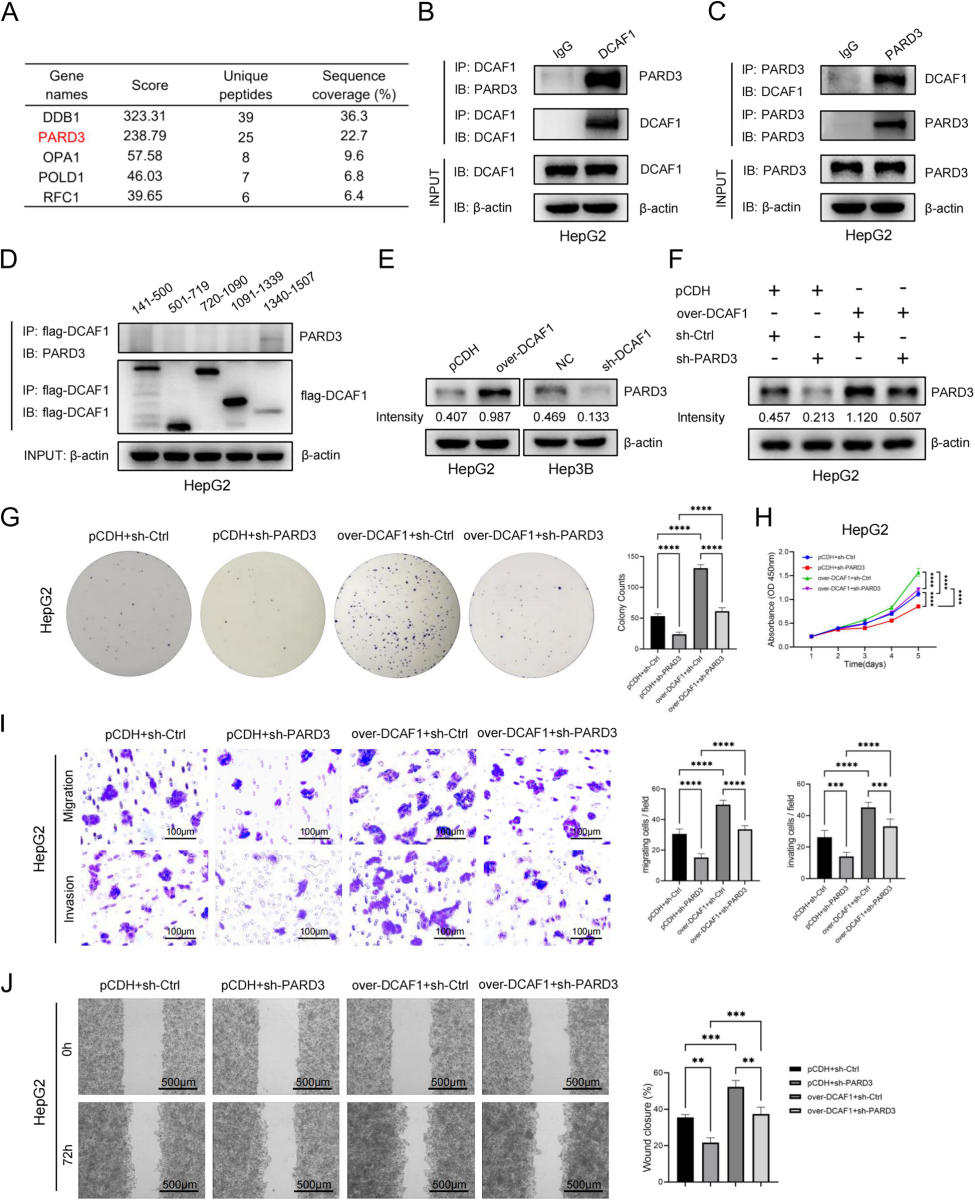
DCAF1 associates with and upregulates PARD3 to promote HCC growth.** A** The top 5 proteins associated with DCAF1 detected by LC–MS/MS. **B, C** Association of DCAF1 and PARD3 verified by coIP. **D** DCAF1 C-terminus containing residues 1339–1507 was found to be responsible for the binding of DCAF1 to PARD3. **E** Western blot results suggested that PARD3 was positively regulated by DCAF1 at the protein level. **F** The expression level of PARD3 in HepG2 cells was verified by Western blot. **G-J** PARD3 partly repressed the promoting effects on HCC cells proliferation and migration caused by DCAF1 overexpression. colony formation assays **G** and CCK8 assays** H** indicated that PARD3 could partly inhibit the promoting effects of DCAF1 overexpression on cell proliferation. Transwell assays **I** and wound healing assays **J** demonstrated that the knockdown of PARD3 partly reversed the promoting effects on cell migration and invasion caused by the overexpression of DCAF1 (Bar = 100μm (**I**), Bar = 500μm (**J**)). Data are shown as the mean ± SD. ** *p* < 0.01, *** *p* < 0.001, **** *p* < 0.0001

To further investigate the molecular mechanism by which DCAF1 regulates HCC progression, proteins that may bind to DCAF1 were extracted by coIP in HepG2 cells overexpressing DCAF1 and then subjected to analysis with liquid chromatography and high-throughput mass spectrometry (LC–MS/MS) (Figure S3A, Supplementary Table 8). Subsequent analysis detected a novel DCAF1-associated protein known as par-3 family cell polarity regulator (PARD3) (Fig. 4A-C, S3B), which has been reported to facilitate HCC progression and predict poor prognosis [14]. Furthermore, to identify the domain of DCAF1 that mediates its interaction with PARD3, we used a series of DCAF1 deletion mutants to conduct immunoprecipitation. The DCAF1 C-terminus containing residues 1339–1507 was found to be responsible for the binding of DCAF1 to PARD3 (Fig. 4D). Interestingly, we observed a reduction in PARD3 protein expression upon DCAF1 knockdown, whereas overexpression of DCAF1 resulted in an elevation of PARD3 protein levels (Fig. 4E). To investigate whether DCAF1 promotes HCC by regulating PARD3, PARD3-shRNA and control vector were transfected into HepG2 cells overexpressing DCAF1 (Fig. 4F). We found that knockdown of PARD3 could partly suppress HCC cell proliferation promoted by DCAF1 overexpression (Fig. 4G, H). Moreover, PARD3 knockdown partly inhibited the enhancement of HepG2 cell migration and invasion by DCAF1 upregulation (Fig. 4I, J). These findings indicate that DCAF1 promotes HCC progression by binding to and upregulating PARD3.

### DCAF1 activates the Akt signaling pathway through upregulating PARD3

**Fig. 5:**
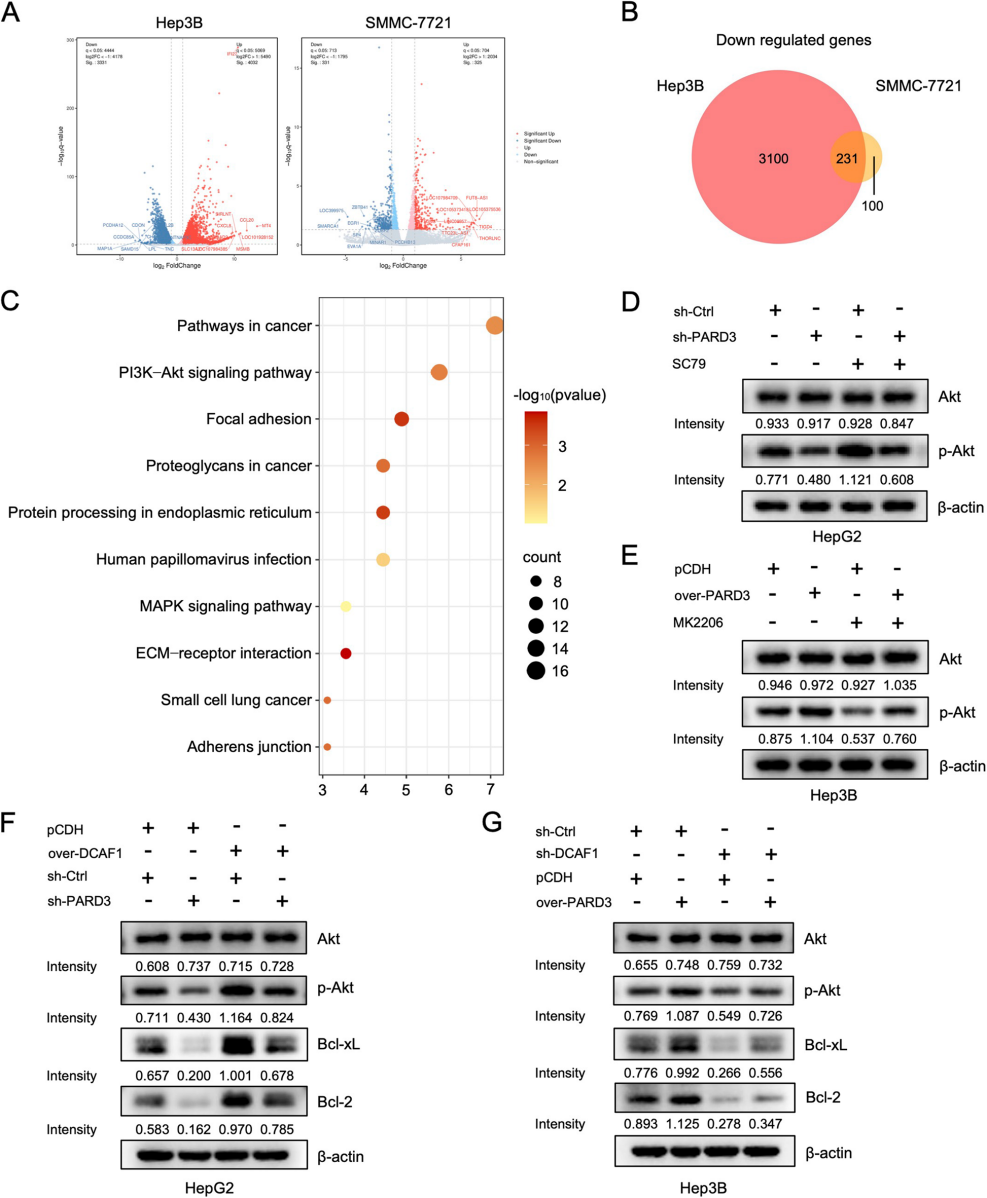
DCAF1 activates the Akt signaling pathway through regulating PARD3. **A** The volcano plots of differentially expressed genes in Hep3B and SMMC-7721 cells transfected with PARD3-shRNA and control vector. Significantly upregulated genes are denoted in red, significantly downregulated genes in blue, and non-significant changes in gray. **B** The Venn grams of downregulated genes in Hep3B and SMMC-7721 cells. **C** KEGG pathway analysis of downregulated genes in Hep3B and SMMC-7721 cells. **D** The protein levels of Akt and p-Akt in HepG2 cells transfected with PARD3-shRNA or control vector were tested by Western blot. **E** The protein levels of Akt and p-Akt in Hep3B cells with PARD3 overexpression or pCDH vector were tested by Western blot. **F** The protein levels of Akt, p-Akt, Bcl-xL and Bcl-2 in HepG2 cells overexpressing DCAF1 transfected with PARD3-shRNA were detected by Western blot. **G** The protein levels of Akt, p-Akt, Bcl-xL and Bcl-2 in Hep3B cells transfected with DCAF1-shRNA overexpressing PARD3 were detected by Western blot

Hep3B and SMMC-7721 cells transfected with PARD3 shRNA or control vector were subjected to RNA sequencing (*n* = 3 for each cell line) in order to explore the key signaling pathway downstream of PARD3. Transcriptomic analysis of Hep3B cells revealed 7363 differentially expressed genes, including 4032 upregulated and 3331 downregulated. The volcano plot displayed the 20 genes with the most significant upregulated and downregulated expression (Fig. 5A). Similarly, transcriptomic sequencing of SMMC-7721 cells identified 656 differentially expressed genes, consisting 325 upregulated and 331 downregulated genes. The volcano plot in Fig. 5A illustrated the 20 genes with the most significant upregulated and downregulated expression. A total of 231 downregulated genes were identified in both Hep3B and SMMC-7721 cells, and were further analyzed using KEGG pathway analysis. The most significantly enriched signaling pathway was the Akt signaling pathway (Fig. 5C). Western blot was employed to verify the impact of PARD3 on Akt activation. The results showed that PARD3 knockdown resulted in a reduction of phosphorylated Akt (p-Akt), which was restored by the Akt activator SC79, while the levels of total Akt remained unchanged (Fig. 5D). Conversely, the Akt inhibitor MK2206 attenuated the elevation of p-Akt induced by PARD3 overexpression (Fig. 5E). These results confirmed the promoting effect of PARD3 on Akt activation. We further examined whether DCAF1 activated the Akt signaling pathway through regulating PARD3. The expression levels of Akt, p-Akt and the downstream proteins were assessed utilizing Western blot. The results, as depicted in Fig. 5F, demonstrated that the overexpression of DCAF1 led to an increase in p-Akt levels, while the levels of total Akt remained unchanged. This observation indicated that the upregulation of DCAF1 promoted Akt activation. Moreover, knockdown of PARD3 effectively abolished the elevation of p-Akt expression (Fig. 5F). Conversely, the downregulation of DCAF1 resulted in a decline in p-Akt protein levels, with no significant change observed in total Akt levels (Fig. 5G). This implied that the downregulation of DCAF1 inhibited the activation of Akt. Notably, the overexpression of PARD3 was able to reinstate p-Akt levels (Fig. 5G). In addition, the antiapoptotic proteins Bcl-2 and Bcl-xL exhibited an increase when DCAF1 was overexpressed, and this elevation was abrogated by PARD3 knockdown (Fig. 5F). Conversely, the expression of Bcl-2 and Bcl-xL decreased when DCAF1 was downregulated, and this effect could be rescued by PARD3 overexpression (Fig. 5G). Collectively, these results strongly suggest that DCAF1 facilitates Akt activation through modulation of PARD3.

### DCAF1 promotes HCC cell proliferation and metastasis via the Akt signaling pathway

**Fig. 6:**
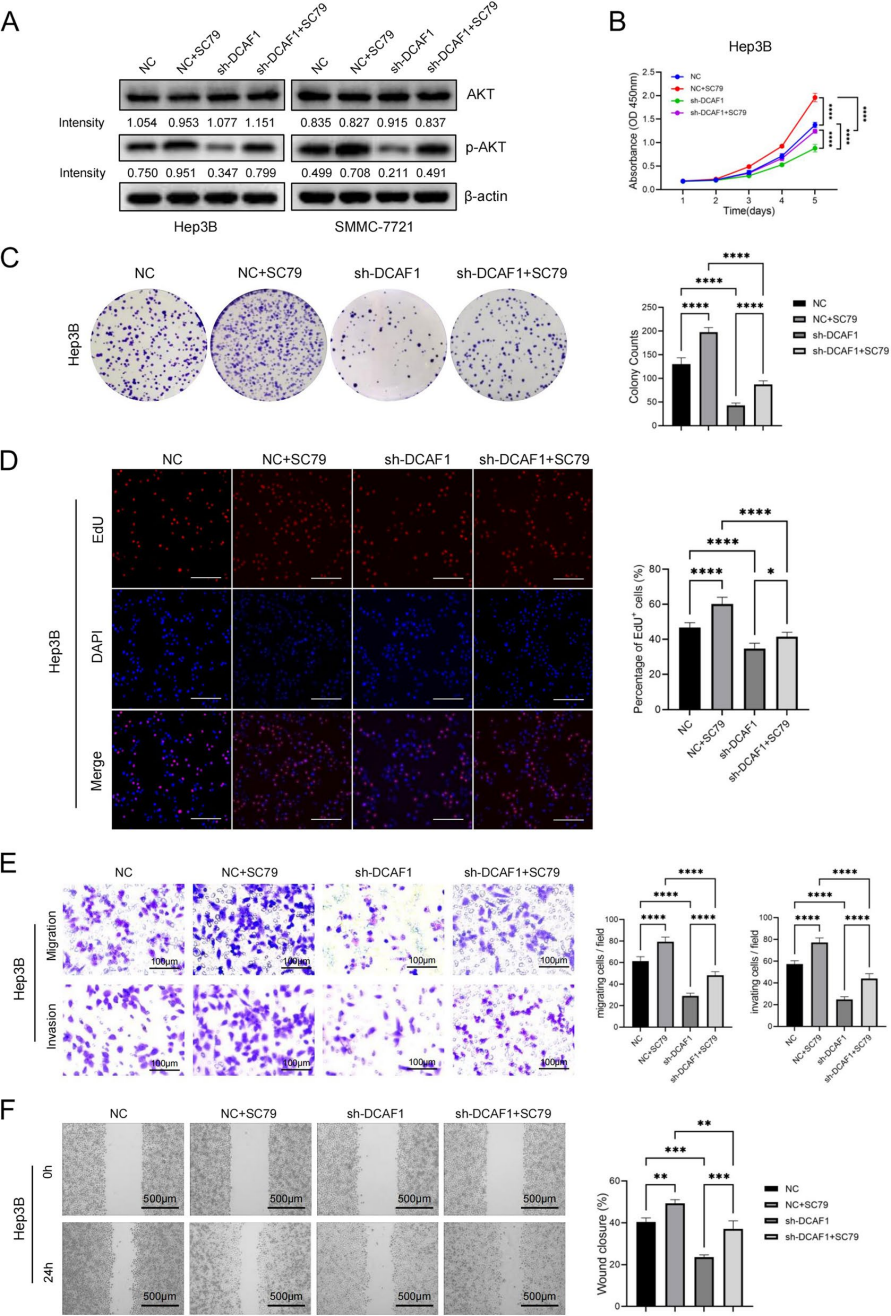
DCAF1 regulates HCC cell proliferation and metastasis by activating the Akt signaling pathway. **A** The protein levels of Akt and p-Akt in Hep3B and SMMC-7721 cells with or without Akt activator were assessed by Western blot. **B**, **D** CCK8 assays **B**, Colony formation assays **C** and EdU assays **D** indicated that the Akt activator could partly rescue the repressive effect caused by DCAF1 knockdown on cell proliferation in Hep3B cells (Bar = 100μm (**D**)). **E**, **F** Transwell assays **E** and wound-healing assays **F** indicated that the Akt activator partly enhanced HCC cell migration and invasion ability in Hep3B cells transfected with DCAF1-shRNA (Bar = 100μm (**E**), Bar = 500μm (**F**)). Data are shown as the mean ± SD. * *p* < 0.05, ** *p* < 0.01, *** *p* < 0.001, **** *p* < 0.0001

Previous investigations have demonstrated that the Akt signaling pathway plays a crucial role in regulating tumor cell proliferation and invasion [20, 21]. To examine the involvement of Akt activation in DCAF1-mediated HCC progression, we treated HCC cells with the Akt activator SC79 or the inhibitor MK2206. The Akt activator increased the phosphorylation of Akt (Fig. 6A) and partially attenuated the cell proliferation rate reduction resulting from DCAF1 knockdown in Hep3B and SMMC-7721 cells (Fig. ​6B-D, Figure ​S3E-G). In addition, pretreatment of HepG2 cells with an Akt inhibitor decreased the phosphorylation of Akt (Figure S3D) and partially abrogated DCAF1-mediated cell proliferation (Figure ​S3E-G). Transwell assays and wound-healing assays indicated that the Akt activator enhanced HCC cell migration and invasion ability in Hep3B and SMMC-7721 cells transfected with DCAF1-shRNA (Fig. 6E-F, S4A-B), while the Akt inhibitor suppressed cell migration and invasion in HepG2 cells overexpressing DCAF1 (Figure S4A-B). These findings provide evidence that the Akt signaling pathway participates in DCAF1-mediated HCC cell proliferation and metastasis.

### The DCAF1/PARD3/Akt axis may provide novel therapeutic approach for HCC

**Fig. 7:**
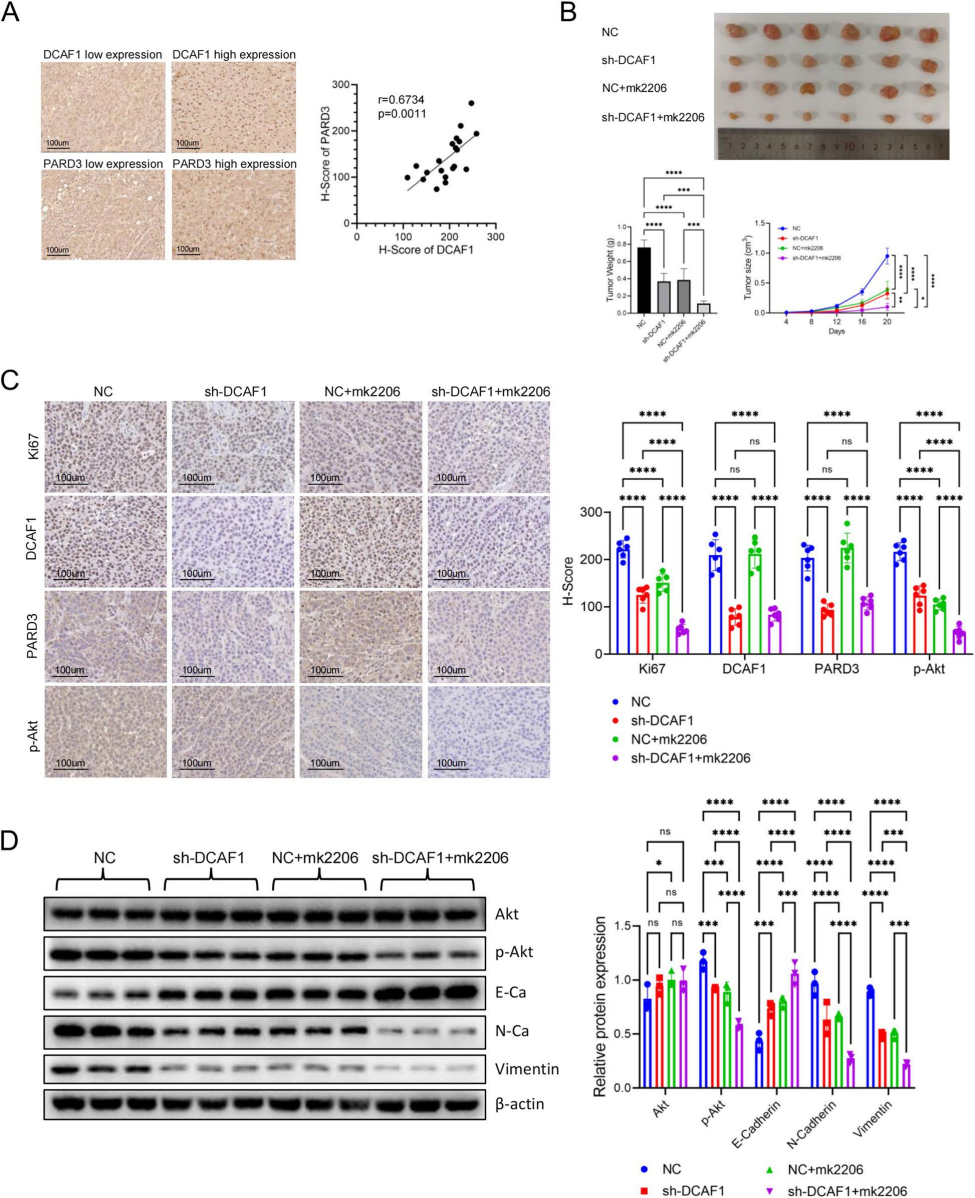
Combined application of DCAF1 knockdown and Akt inhibitor synergistically suppresses tumor growth. **A** PARD3 and DCAF1 in tissue chips from HCC patients (*n* = 20) were detected by IHC (Bar = 100μm).** B** Hep3B cells with DCAF1 knockdown, Akt inhibitor treatment, or a combination of both were transplanted on nude mice. Tumor volumes and tumor weights were recorded. **C** Detection of Ki67, DCAF1, PARD3 and p-Akt in the tumor tissues by IHC staining (Bar = 100μm). **D** Assessment of Akt, p-Akt and EMT marker proteins in xenografts by Western blot. * *p* < 0.05, ** *p* < 0.01, *** *p* < 0.001, **** *p* < 0.0001

The expression levels of DCAF1 and PARD3 in the tumor tissues of HCC patients (*n* = 20) were detected by IHC (Fig. 7A). The results showed a significant positive correlation of DCAF1 and PARD3 expression. Furthermore, a subcutaneous tumor model was generated to investigate the suppressive effects of the Akt inhibitor combined with DCAF1 knockdown on tumors. Hep3B cells with DCAF1 knockdown, Akt inhibitor treatment, or a combination of both were transplanted on nude mice. The tumor weights and sizes of each group were recorded (Fig. 7B). IHC assays were then performed to detect Ki67, DCAF1, PARD3 and p-Akt expression, followed by the calculation of H-Scores (Fig. 7C). The results indicate that both inhibiting DCAF1 and using the Akt inhibitor effectively hinder tumor growth, and their combined application synergistically suppress tumor growth. Additionally, Western blot was employed to assess the protein expression of Akt, p-Akt and EMT markers in xenografts (Fig. 7D). The results showed that both DCAF1 knockdown and Akt inhibitor reduced the expression of N-Cadherin and Vimentin, while increasing E-Cadherin expression. Combining Akt inhibitor with DCAF1 knockdown further decreased mesenchymal markers, indicating a greater inhibition of EMT. These results suggest that DCAF1/PARD3/Akt axis presents a potentially effective therapeutic strategy for HCC.

## Discussion

HCC is a major type of liver cancer with unfavorable clinical outcomes. Given the limited efficacy of existing therapies, there is a pressing need to identify novel molecular targets to enhance the prognosis of HCC. In our investigation, we observed a correlation between DCAF1 and HCC overall survival, as well as clinicopathological features. DCAF1 exhibited upregulation in both HCC tissues and cell lines. Knockdown of DCAF1 exerted inhibitory effects on HCC proliferation and metastasis by suppressing Akt activation, whereas overexpression of DCAF1 resulted in the opposite outcomes through activation of Akt signaling pathway. In addition, the combined application of Akt inhibitor and DCAF1-shRNA could further inhibit the proliferation of subcutaneous xenograft models.

DCAF1-induced Akt activation depends on PARD3, as detected by Western blot. Previous studies have reported that PARD3 can regulate cell polarity, cell division, and cell migration [13]. PARD3 was enriched in HCC primitive tumour cells and its overexpression enhanced tumour-initiating cell (TICs). Overexpression of PARD3 maintained the self-renewal ability of the CD133 + TIC population within hepatocellular carcinoma (HCC) cells and promoted the in vitro and in vivo tumorigenicity of CD133 + TICs. Mechanistically, PARD3 interacted with aPKC to further activate SHH signalling and downstream stemness-related genes [15]. In the present investigation, the expression levels of PARD3 and DCAF1 were positively correlated in HCC tissues (Fig. 7A). PARD3 could bind to the C-terminus containing residues 1339–1507 of DCAF1 (Fig. 4D). Knockdown of PARD3 partly suppressed the increased proliferation and migration ability caused by DCAF1 overexpression.

Akt signaling pathway is one of the crucial signaling pathways responsible for PARD3-mediated cell survival [22, 23], consistent with our RNA sequencing and Western blot results (Fig. 5A-E). Cancers characterized by activated Akt signaling have been demonstrated to exhibit increased aggressiveness [24, 25], and Akt activation has been identified as a significant risk factor for earlier recurrence and poor prognosis in patients with liver cancer [26]. Considering the association between PARD3 and DCAF1, we explored whether DCAF1 affected the Akt signaling pathway in HCC. Our results showed that p-Akt was upregulated due to DCAF1 overexpression, and the Akt inhibitor partially abrogated DCAF1-mediated cell proliferation and invasion, indicating that DCAF1 promotes HCC progression by activating the Akt signaling pathway.

Given the involvement of the Akt signaling pathway in numerous crucial cellular processes, the targeting of this pathway often leads to adverse events, prompting early treatment termination or study cancellation [27]. Moreover, the emergence of treatment resistance following Akt pathway inhibition has been observed [28]. Combination therapies are increasingly recognized as crucial components in new drug research and development, particularly in the field of cancer [29, 30]. Our findings indicate that the combined use of DCAF1-shRNA can significantly enhance the efficacy of an Akt inhibitor. In a clinical context, patients with low DCAF1 expression may derive greater benefits from Akt inhibitors such as Ipatasertib or Capivasertib. Consequently, it is imperative to develop new drugs or combination therapies to enhance the tolerability and efficiency of Akt inhibitors.

## Conclusion

On the whole, our investigation revealed that DCAF1 could promote HCC progression by activating the Akt signaling pathway through binding to PARD3 and enhancing its expression. Our study illustrates that DCAF1 plays a crucial role in HCC development and the DCAF1/PARD3/Akt axis presents a potentially effective therapeutic strategy for HCC.

### Supplementary Information

**Supplementary Material 1.**

## Data Availability

The datasets used and/or analyzed during the current study are available from the corresponding author on reasonable request.

## Abbreviations

HCC: Hepatocellular carcinoma
DCAFs: DDB1 and CUL4 associated factors
DCAF1: DDB1 and CUL4 associated factor 1
Akt: Akt serine/threonine kinase
p-Akt: Phosphorylated Akt
PARD3: Par-3 family cell polarity regulator
qRT‒PCR: Quantitative real-time PCR
DMEM: Dulbecco’s modified Eagle’s medium
coIP: Coimmunoprecipitation
IF: Immunofluorescence
IHC: Immunohistochemistry
DEGs: Differentially expressed genes
EMT: Epithelial-mesenchymal transition
LC–MS/MS: Liquid chromatography and high-throughput mass spectrometry

## References

[1] Siegel RL, Miller KD, Wagle NS, Jemal A (2023). Cancer statistics, 2023. CA Cancer J Clin.

[2] Llovet JM, Kelley RK, Villanueva A, Singal AG, Pikarsky E, Roayaie S (2021). Hepatocellular carcinoma. Nat Rev Dis Primers.

[3] Le Rouzic E, Belaïdouni N, Estrabaud E, Morel M, Rain JC, Transy C (2007). HIV1 Vpr arrests the cell cycle by recruiting DCAF1/VprBP, a receptor of the Cul4-DDB1 ubiquitin ligase. Cell Cycle.

[4] Zhou Z, Song X, Wavelet CM, Wan Y (2020). Cullin 4-DCAF Proteins in Tumorigenesis. Adv Exp Med Biol.

[5] Han XR, Sasaki N, Jackson SC, Wang P, Li Z, Smith MD, et al. CRL4(DCAF1/VprBP) E3 ubiquitin ligase controls ribosome biogenesis, cell proliferation, and development. Sci Adv. 2020;6(51):eabd6078. 10.1126/sciadv.abd6078 PMC11206221 33355139

[6] Hrecka K, Hao C, Shun MC, Kaur S, Swanson SK, Florens L (2016). HIV-1 and HIV-2 exhibit divergent interactions with HLTF and UNG2 DNA repair proteins. Proc Natl Acad Sci U S A.

[7] Yurkovetskiy L, Guney MH, Kim K, Goh SL, McCauley S, Dauphin A (2018). Primate immunodeficiency virus proteins Vpx and Vpr counteract transcriptional repression of proviruses by the HUSH complex. Nat Microbiol.

[8] Liu J, Yuan B, Cao J, Luo H, Gu S, Zhang M (2021). AMBRA1 Promotes TGFβ Signaling via Nonproteolytic Polyubiquitylation of Smad4. Cancer Res.

[9] Tang J, Wu Z, Wang X, Hou Y, Bai Y, Tian Y. Hypoxia-Regulated lncRNA USP2-AS1 Drives Head and Neck Squamous Cell Carcinoma Progression. Cells. 2022;11(21):3407. 10.3390/cells11213407 PMC9655520 36359803

[10] Wang X, Arceci A, Bird K, Mills CA, Choudhury R, Kernan JL, et al. VprBP/DCAF1 Regulates the Degradation and Nonproteolytic Activation of the Cell Cycle Transcription Factor FoxM1. Mol Cell Biol. 2017;37(13):e00609-16. 10.1128/MCB.00609-16 PMC5472828 28416635

[11] Ren W, Shen S, Sun Z, Shu P, Shen X, Bu C (2016). Jak-STAT3 pathway triggers DICER1 for proteasomal degradation by ubiquitin ligase complex of CUL4A(DCAF1) to promote colon cancer development. Cancer Lett.

[12] Poulose N, Forsythe N, Polonski A, Gregg G, Maguire S, Fuchs M (2022). VPRBP functions downstream of the androgen receptor and OGT to Restrict p53 activation in prostate cancer. Mol Cancer Res.

[13] Atashrazm F, Ellis S (2021). The polarity protein PARD3 and cancer. Oncogene.

[14] Li S, Huang J, Yang F, Zeng H, Tong Y, Li K (2021). High expression of PARD3 predicts poor prognosis in hepatocellular carcinoma. Sci Rep.

[15] Wu J, Tan HY, Chan YT, Lu Y, Feng Z, Yuan H (2024). PARD3 drives tumorigenesis through activating Sonic Hedgehog signalling in tumour-initiating cells in liver cancer. J Exp Clin Cancer Res.

[16] Gou H, Wong CC, Chen H, Shang H, Su H, Zhai J (2023). TRIP6 disrupts tight junctions to promote metastasis and drug resistance and is a therapeutic target in colorectal cancer. Cancer Lett.

[17] Sun X, Jin Z, Song X, Wang J, Li Y, Qian X (2015). Evaluation of KIF23 variant 1 expression and relevance as a novel prognostic factor in patients with hepatocellular carcinoma. BMC Cancer.

[18] Mittal V (2018). Epithelial mesenchymal transition in tumor metastasis. Annu Rev Pathol.

[19] Yang Y, Ren P, Liu X, Sun X, Zhang C, Du X (2022). PPP1R26 drives hepatocellular carcinoma progression by controlling glycolysis and epithelial-mesenchymal transition. J Exp Clin Cancer Res.

[20] Yang J, Huang Y, Song M, Pan Q, Zhao J, He J (2022). SPC25 promotes proliferation and stemness of hepatocellular carcinoma cells via the DNA-PK/AKT/Notch1 signaling pathway. Int J Biol Sci.

[21] Sun F, Wang J, Sun Q, Li F, Gao H, Xu L (2019). Interleukin-8 promotes integrin β3 upregulation and cell invasion through PI3K/Akt pathway in hepatocellular carcinoma. J Exp Clin Cancer Res.

[22] Ahmed SM, Macara IG (2017). The Par3 polarity protein is an exocyst receptor essential for mammary cell survival. Nat Commun.

[23] Gao W, Guo H, Niu M, Zheng X, Zhang Y, Xue X (2020). circPARD3 drives malignant progression and chemoresistance of laryngeal squamous cell carcinoma by inhibiting autophagy through the PRKCI-Akt-mTOR pathway. Mol Cancer.

[24] Bamodu OA, Chang HL, Ong JR, Lee WH, Yeh CT, Tsai JT. Elevated PDK1 Expression Drives PI3K/AKT/MTOR Signaling Promotes Radiation-Resistant and Dedifferentiated Phenotype of Hepatocellular Carcinoma. Cells. 2020;9(3):746. 10.3390/cells9030746 PMC7140693 32197467

[25] Zhang F, Li K, Pan M, Li W, Wu J, Li M (2018). miR-589 promotes gastric cancer aggressiveness by a LIFR-PI3K/AKT-c-Jun regulatory feedback loop. J Exp Clin Cancer Res.

[26] Nakanishi K, Sakamoto M, Yamasaki S, Todo S, Hirohashi S (2005). Akt phosphorylation is a risk factor for early disease recurrence and poor prognosis in hepatocellular carcinoma. Cancer.

[27] Bang J, Jun M, Lee S, Moon H, Ro SW. Targeting EGFR/PI3K/AKT/mTOR Signaling in Hepatocellular Carcinoma. Pharmaceutics. 2023;15(8):2130. 10.3390/pharmaceutics15082130 PMC10458925 37631344

[28] Formisano L, Napolitano F, Rosa R, D'Amato V, Servetto A, Marciano R (2020). Mechanisms of resistance to mTOR inhibitors. Crit Rev Oncol Hematol.

[29] Jin H, Wang L, Bernards R (2023). Rational combinations of targeted cancer therapies: background, advances and challenges. Nat Rev Drug Discov.

[30] Wang Y, Minden A. Current Molecular Combination Therapies Used for the Treatment of Breast Cancer. Int J Mol Sci. 2022;23(19):11046. 10.3390/ijms231911046 PMC9569555 36232349

