# Supplementary material for: DCAF1 interacts with PARD3 to promote hepatocellular carcinoma progression and metastasis by activating the Akt signaling pathway
Source: J Exp Clin Cancer Res. 2024 May 6;43:136. doi: 10.1186/s13046-024-03055-2 (PMC11071249; doi:10.1186/s13046-024-03055-2)
Supplement: Supplementary file 1 — Supplementary Material 1. [file 13046_2024_3055_MOESM1_ESM.docx]

**
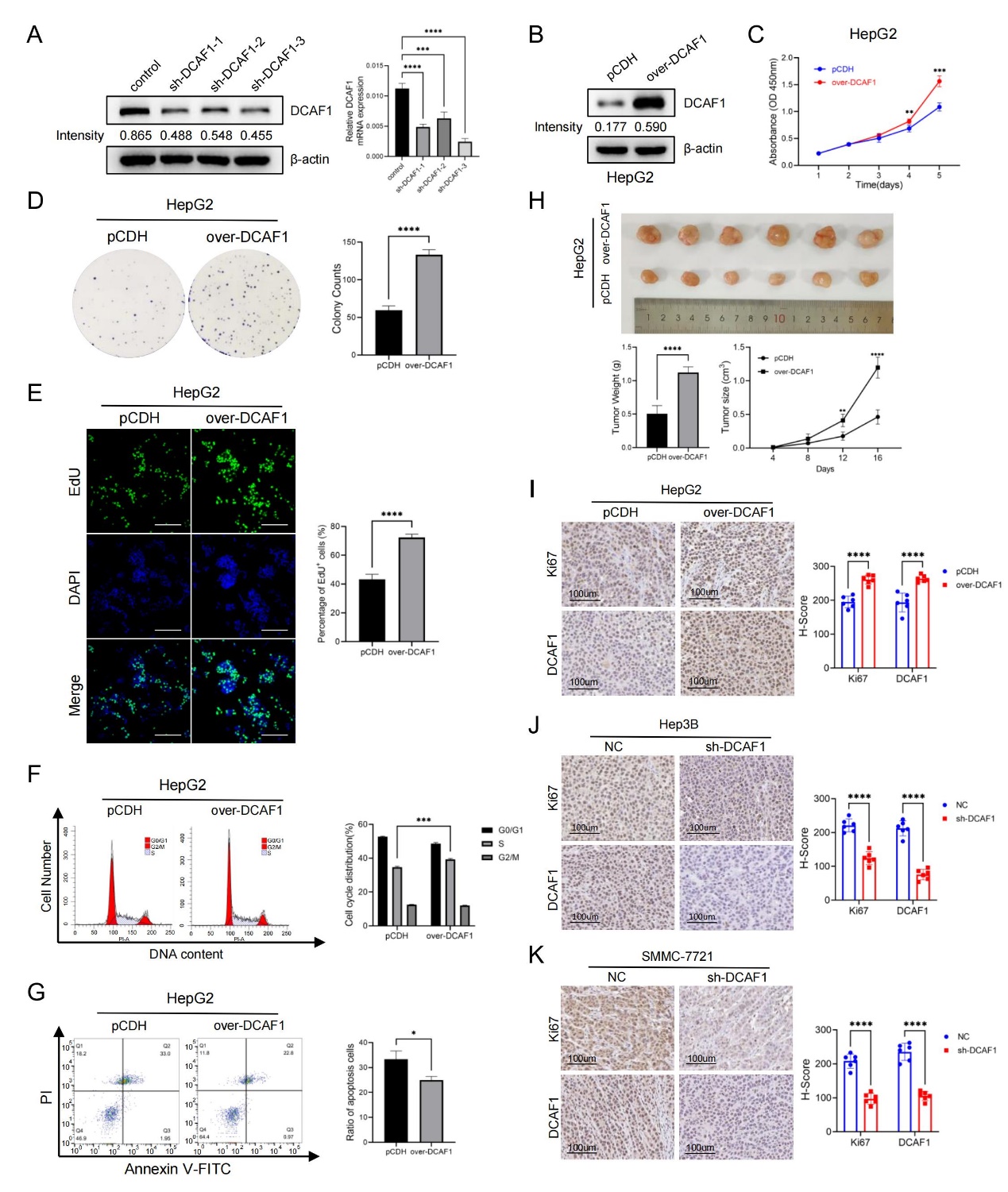
**

**Figure S1** DCAF1 promotes HCC growth in vitro and in vivo. **A**-**B** The efficacy of DCAF1 knockdown in Hep3B cells and overexpression in HepG2 cells were verified by Western blot. **C** CCK8 assays in HepG2 cells after overexpression of DCAF1. **D** The colony formation results of HepG2 cells after DCAF1 overexpression. **E** EdU assay results of HepG2 cells after overexpression of DCAF1 (Bar=100μm). **F** Cell cycle analysis of HepG2 cells was conducted by flow cytometry. **G** The cell apoptosis ratio of HepG2 cells was detected by flow cytometry using an Annexin V-FITC/PI staining kit. **H** HepG2 cells with DCAF1 overexpression were transplanted on nude mice, and tumor volumes and tumor weights were recorded. **I-K** Representative IHC images of Ki67 and DCAF1 in the HepG2 (**I**)、Hep3B (**J**)、SMMC-7721 (**K**) subcutaneous xenograft model (Bar=100μm). Data are shown as the mean ± SD. * *p* < 0.05, ** *p* < 0.01, *** *p* < 0.001, **** *p* < 0.0001.


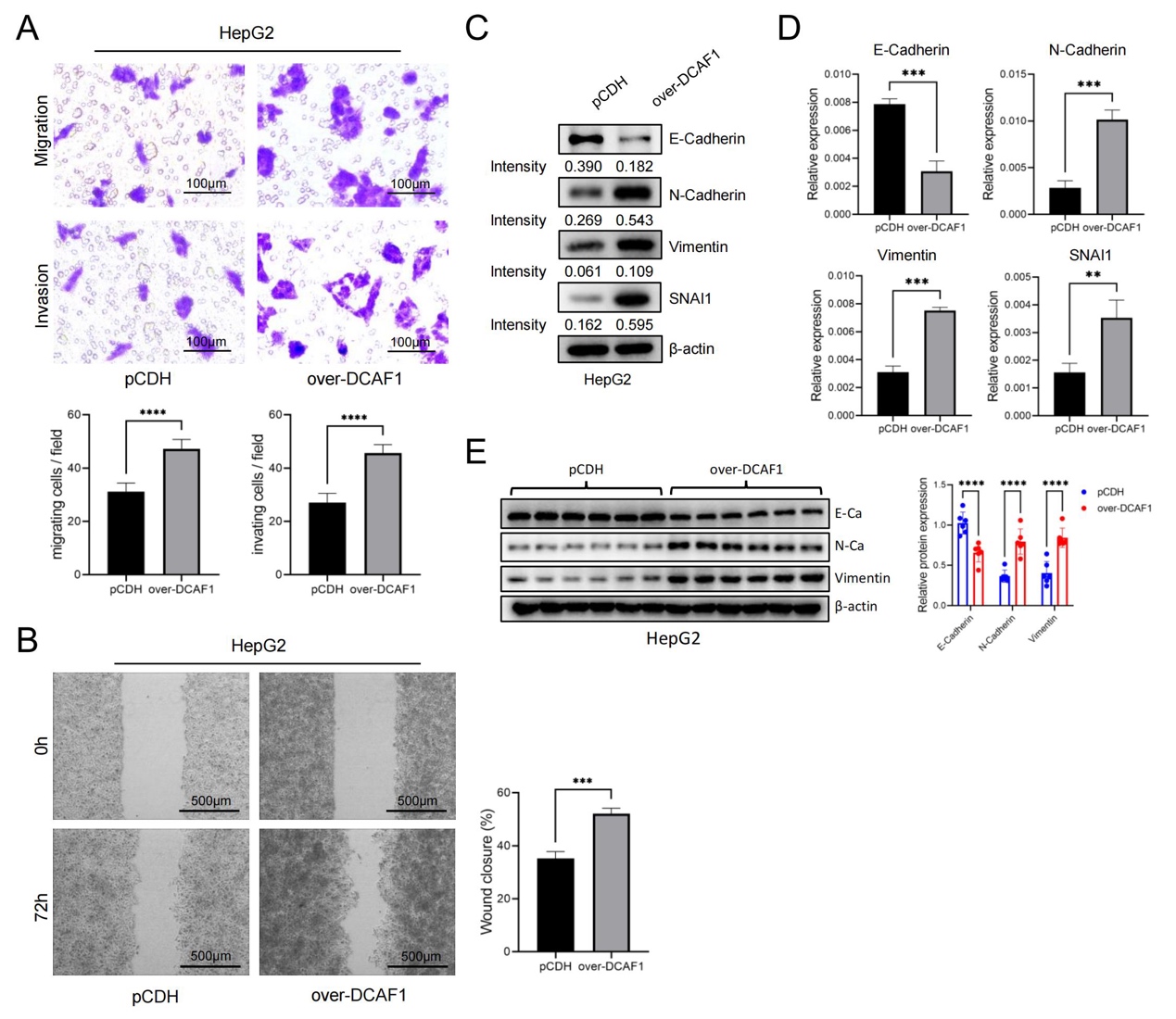


**Figure S2** DCAF1 regulates cell migration and invasion in HepG2 cells and activates EMT. **A** Representative image of migrated and invaded HepG2 cells after DCAF1 overexpression (Bar=100μm). **B** Wound healing assays were performed to assess the effect of overexpression of DCAF1 on cell motility in HepG2 cells (Bar=500μm). **C** The expression levels of EMT markers in HepG2 cells after DCAF1 overexpression were detected by Western blot. **D** The mRNA expression levels of EMT markers in HepG2 cells after DCAF1 overexpression were detected by qRT‒PCR. **E** The expression of EMT marker proteins in HepG2 subcutaneous xenografts were assessed by Western blot. Data are shown as the mean ± SD. * *p* < 0.05, ** *p* < 0.01, *** *p* < 0.001, **** *p* < 0.0001.


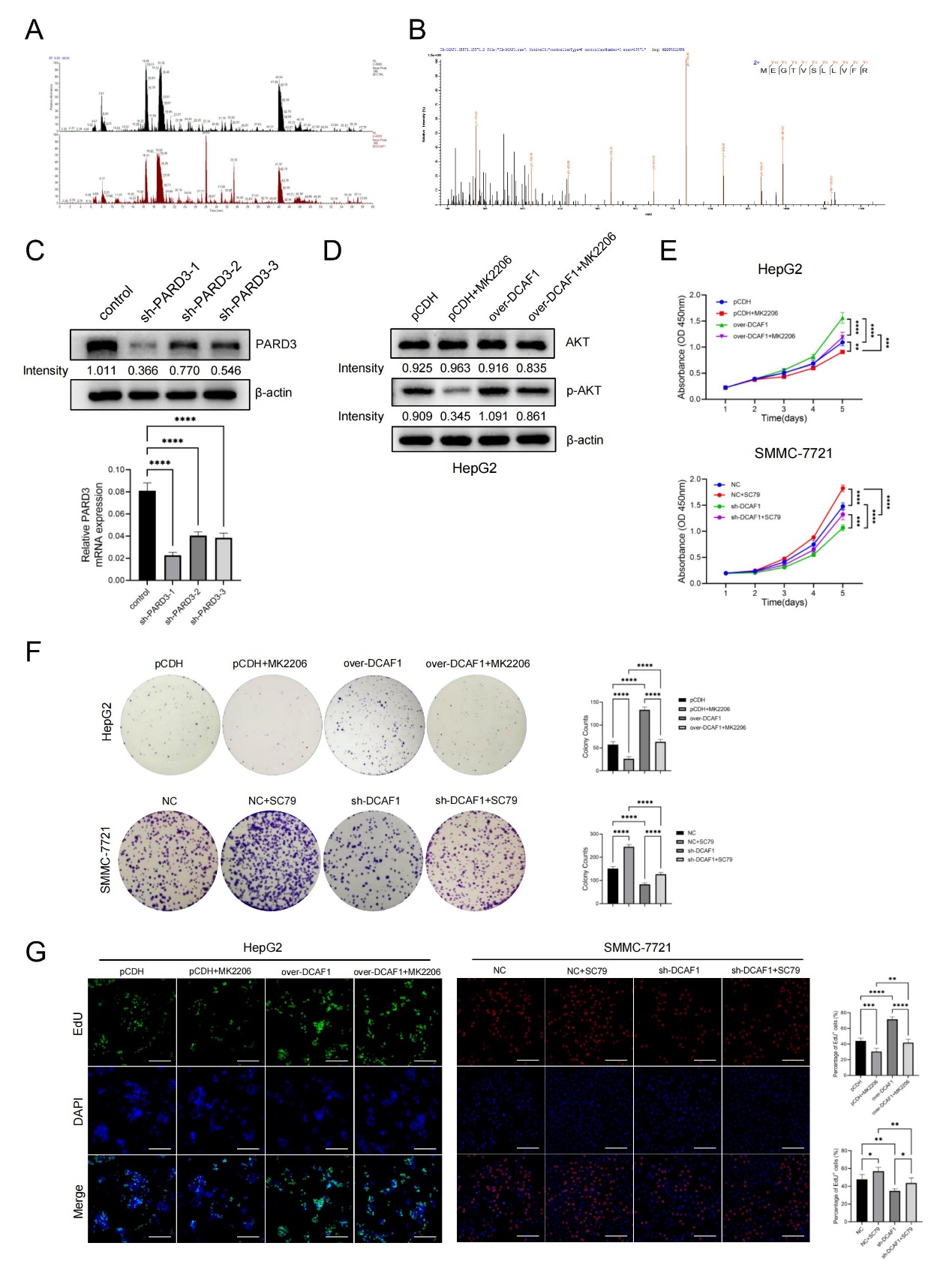


**Figure S3** DCAF1 binds to PARD3 thus activating the Akt signaling pathway to promote HCC cell proliferation. **A-B** Mass spectrometry analysis. **A** The base peaks of mass spectrometry. **B** The mass spectrogram of a PARD3 unique peptide. **C** The efficacy of PARD3 knockdown in HepG2 cells verified by western blot and RT‒qPCR. **D** The protein levels of Akt and p-Akt in HepG2 cells with or without the Akt inhibitor were detected by Western blot. **E-G** CCK8 assays (**E)**, colony formation assays (**F)** and EdU assays (**G)** indicated that the Akt activator could partly rescue the repressive effect on cell proliferation caused by DCAF1 knockdown in SMMC-7721 cells, while the Akt inhibitor could partly suppress the enhancing effect of DCAF1 overexpression on the proliferation of HepG2 cells (Bar=100μm). Data are shown as the mean ± SD. * *p* < 0.05, ** *p* < 0.01, *** *p* < 0.001, **** *p* < 0.0001.


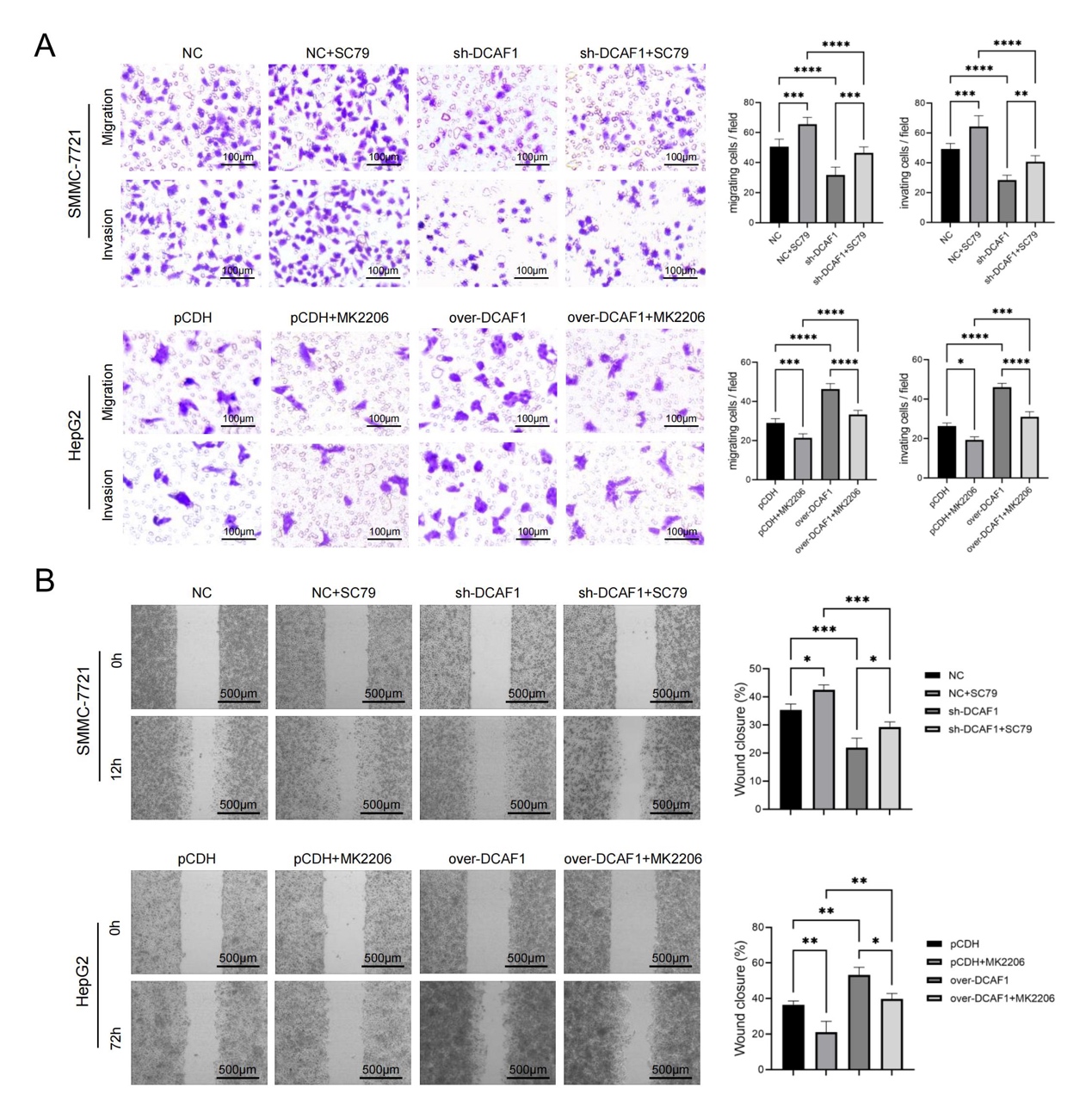


**Figure S4** DCAF1 promotes HCC cell metastasis by activating the Akt signaling pathway. **A-B** Transwell assays **A** and wound-healing assays **B** indicated that the Akt activator could partly rescue the repressive effect on cell migration and invasion caused by DCAF1 knockdown in SMMC-7721 cells, while the Akt inhibitor could partly suppress the enhancing effect of DCAF1 overexpression on the migration and invasion of HepG2 cells (Bar=100μm (**A**), Bar=500μm (**B**)). Data are shown as the mean ± SD. * *p* < 0.05, ** *p* < 0.01, *** *p* < 0.001, **** *p* < 0.0001.

**Supplementary Table 1 Sequences of shRNAs**

| Plasmid name | Target sequence |
| --- | --- |
| sh-DCAF1-1 | GCAGTGACCATGTCAAGTT |
| sh-DCAF1-2 | GGTGTCAGGAAAACCACTT |
| sh-DCAF1-3 | GCGCCAATAAACTTTACGTCA |
| sh-PARD3-1 | GGAATCCACGTAGTGCCTTTC |
| sh-PARD3-2 | GCACCTCAGAATGTATTTAGT |
| sh-PARD3-3 | GGGCAAATCCCAAGAGGAAGT |

**Supplementary Table 2 Design of DCAF1 deletion mutants**

| Designed segments | | Actual segments | | |
| --- | --- | --- | --- | --- |
| Name | Length | Type | Region | Structural domain |
| DCAF1(1-140aa) | 140 |  |  |  |
| DCAF1(141-500aa) | 360 | Region | 141-500 | Protein kinase-like |
|  |  | Region | 242-288 | Disordered Automatic Annotation |
| DCAF1(501-719aa) | 249 | Domain | 562-593 | Chromo |
| DCAF1(720-1090aa) | 371 | Domain | 846-878 | LisHPROSITE-ProRule Annotation |
|  |  | Region | 917-947 | Disordered Automatic Annotation |
| DCAF1(1091-1339aa) | 248 | Repeat | 1091-1130 | WD 1 |
|  |  | Region | 1091-1290 | WD repeat-like region |
|  |  | Repeat | 1133-1174 | WD 2 |
|  |  | Repeat | 1176-1213 | WD 3 |
|  |  | Repeat | 1215-1247 | WD 4 |
|  |  | Motif | 1242-1249 | DWD box 1 |
|  |  | Repeat | 1248-1290 | WD 5 |
|  |  | Motif | 1278-1285 | DWD box 2 |
| DCAF1(1340-1507aa) | 168 | Region | 1393-1507 | Disordered Automatic Annotation |
|  |  | Region | 1418-1507 | Interaction with NF2 |

**Supplementary Table 3 Primer names and sequences**

| **Primer name** | **Sequence** |
| --- | --- |
| PARD3- F | CAGGTGCATCGCTTGGAAC |
| PARD3- R | GCTGAGACATTGTTGGTGCC |
| DCAF1- F | GGAGGGAATTGTCGAGAATCTTT |
| DCAF1- R | GTTGGCAGCAATGTCTTGATTT |
| E-cadherin-F | ATTTTTCCCTCGACACCCGAT |
| E-cadherin-R | TCCCAGGCGTAGACCAAGA |
| N-cadherin-F | AGCCAACCTTAACTGAGGAGT |
| N-cadherin-R | GGCAAGTTGATTGGAGGGATG |
| Vimentin-F | AGTCCACTGAGTACCGGAGAC |
| Vimentin-R | CATTTCACGCATCTGGCGTTC |
| SNAI1-F | TCGGAAGCCTAACTACAGCGA |
| SNAI1-R | AGATGAGCATTGGCAGCGAG |

**Supplementary Table 4 Antibodies for Western blot**

| **Antibody** | **Order** | **Catalog** | **Dilution** | **Manufacturer** |
| --- | --- | --- | --- | --- |
| anti-DCAF1 | Primary | 11612-1-AP | 1:1000 | Proteintech |
| anti-PARD3 | Primary | 11085-1-AP | 1:1000 | Proteintech |
| anti-Akt | Primary | 9272 | 1:4000 | CST |
| anti-p-Akt | Primary | 4060 | 1:1000 | CST |
| anti-Bcl-xL | Primary | 2764 | 1:1000 | CST |
| anti-Bcl-2 | Primary | 3498 | 1:1000 | CST |
| anti-E-Cadherin | Primary | 3195 | 1:1000 | CST |
| anti-N-Cadherin | Primary | A19083 Abclonal | 1:1000 | CST |
| anti-Vimentin  anti-SNAI1  anti-β-actin | Primary | 60330-1-Ig | 1:20000 | Proteintech |
| anti-SNAI1 | Primary | 13099-1-AP | 1:1000 | Proteintech |
| anti-β-actin | Primary | 66009-1-Ig | 1:20000 | Proteintech |
| anti-flag | Primary | 66008-4-Ig | 1:5000 | Proteintech |
| anti-Mouse | Secondary | 7076 | 1:2000 | CST |
| anti-Rabbit | Secondary | 7074 | 1:2000 | CST |

**Supplementary Table 5 Antibodies for Coimmunoprecipitation**

| **Antibody** | **Catalog** | **Dilution** | **Manufacturer** |
| --- | --- | --- | --- |
| anti-DCAF1 | 11612-1-AP | 4 ug for 1ml protein lysate | Proteintech |
| anti-PARD3 | 11085-1-AP | 4 ug for 1ml protein lysate | Proteintech |
| anti-flag | 20543-1-AP | 4 ug for 1ml protein lysate | Proteintech |
| Rabbit (DA1E) mAb IgG XP^®^ Isotype Control | 3900 | 4 ug for 1ml protein lysate | CST |

**Supplementary Table 6 Antibodies for Immunofluorescence**

| **Antibody** | **Order** | **Catalog** | **Dilution** | **Manufacturer** |
| --- | --- | --- | --- | --- |
| anti-DCAF1 | Primary | 11612-1-AP | 1:200 | Proteintech |
| Goat anti-Rabbit IgG (H+L) Cross-Adsorbed Secondary Antibody, Alexa Fluor™ 488 | Secondary | A-11008 | 1:200 | Invitrogen |

**Supplementary Table 7 Antibodies for IHC**

| **Antibody** | **Order**  **Primary**  **Primary**  **Primary**  **Primary** | **Catalog** | **Dilution** | **Manufacturer** |
| --- | --- | --- | --- | --- |
| anti-DCAF1 | Primary  Primary  Primary  Primary | 11612-1-AP | 1:200 | Proteintech |
| anti-PARD3 | Primary | 11085-1-AP | 1:200 | Proteintech |
| anti-p-Akt | Primary | 4060 | 1:200 | CST |
| anti-Ki67 | Primary | 12202 | 1:200 | CST |
| anti-Rabbit/Mouse | Secondary | SP KIT-B3 | Directedly used used | MAXIM |

**Supplementary Table 8 Proteins identified by mass spectrometry that may interact with DCAF1**

| **Protein IDs** | **Protein names** | **Gene names** | **Score** | **Sequence coverage [%]** | **Unique peptides** | **Fasta headers** |
| --- | --- | --- | --- | --- | --- | --- |
| Q16531 | DNA damage-binding protein 1 | DDB1 | 323.31 | 36.3 | 39 | sp\|Q16531\|DDB1_HUMAN DNA damage-binding protein 1 OS=Homo sapiens OX=9606 GN=DDB1 PE=1 SV=1 |
| Q8TEW0 | Partitioning defective 3 homolog | PARD3 | 238.79 | 22.7 | 25 | sp\|Q8TEW0\|PARD3_HUMAN Partitioning defective 3 homolog OS=Homo sapiens OX=9606 GN=PARD3 PE=1 SV=2 |
| O60313 | Dynamin-like 120 kDa protein, mitochondrial | OPA1 | 57.58 | 9.6 | 8 | sp\|O60313\|OPA1_HUMAN Dynamin-like 120 kDa protein, mitochondrial OS=Homo sapiens OX=9606 GN=OPA1 PE=1 SV=3 |
| P28340 | DNA polymerase delta catalytic subunit | POLD1 | 46.027 | 6.8 | 7 | sp\|P28340\|DPOD1_HUMAN DNA polymerase delta catalytic subunit OS=Homo sapiens OX=9606 GN=POLD1 PE=1 SV=2 |
| P35251 | Replication factor C subunit 1 | RFC1 | 39.649 | 6.4 | 6 | sp\|P35251\|RFC1_HUMAN Replication factor C subunit 1 OS=Homo sapiens OX=9606 GN=RFC1 PE=1 SV=4 |
| P19474 | E3 ubiquitin-protein ligase TRIM21 | TRIM21 | 32.745 | 14.1 | 5 | sp\|P19474\|RO52_HUMAN E3 ubiquitin-protein ligase TRIM21 OS=Homo sapiens OX=9606 GN=TRIM21 PE=1 SV=1 |
| Q9BW61 | DET1- and DDB1-associated protein 1 | DDA1 | 29.953 | 44.1 | 4 | sp\|Q9BW61\|DDA1_HUMAN DET1- and DDB1-associated protein 1 OS=Homo sapiens OX=9606 GN=DDA1 PE=1 SV=1 |
| Q96N67;Q96HP0 | Dedicator of cytokinesis protein 7 | DOCK7 | 25.668 | 2 | 4 | sp\|Q96N67\|DOCK7_HUMAN Dedicator of cytokinesis protein 7 OS=Homo sapiens OX=9606 GN=DOCK7 PE=1 SV=4 |
| Q6UN15 | Pre-mRNA 3-end-processing factor FIP1 | FIP1L1 | 18.763 | 4.9 | 2 | sp\|Q6UN15\|FIP1_HUMAN Pre-mRNA 3-end-processing factor FIP1 OS=Homo sapiens OX=9606 GN=FIP1L1 PE=1 SV=1 |
| Q9UKM9 | RNA-binding protein Raly | RALY | 18.17 | 9.5 | 3 | sp\|Q9UKM9\|RALY_HUMAN RNA-binding protein Raly OS=Homo sapiens OX=9606 GN=RALY PE=1 SV=1 |
| P47929 | Galectin-7 | LGALS7B | 17.111 | 16.9 | 2 | sp\|P47929\|LEG7_HUMAN Galectin-7 OS=Homo sapiens OX=9606 GN=LGALS7B PE=1 SV=2 |
| O75127 | Pentatricopeptide repeat-containing protein 1, mitochondrial | PTCD1 | 14.663 | 2.7 | 2 | sp\|O75127\|PTCD1_HUMAN Pentatricopeptide repeat-containing protein 1, mitochondrial OS=Homo sapiens OX=9606 GN=PTCD1 PE=1 SV=2 |
| Q9BU76 | Multiple myeloma tumor-associated protein 2 | MMTAG2 | 12.723 | 8 | 2 | sp\|Q9BU76\|MMTA2_HUMAN Multiple myeloma tumor-associated protein 2 OS=Homo sapiens OX=9606 GN=MMTAG2 PE=1 SV=1 |
| Q9C0J8 | pre-mRNA 3 end processing protein WDR33 | WDR33 | 12.446 | 1.9 | 2 | sp\|Q9C0J8\|WDR33_HUMAN pre-mRNA 3 end processing protein WDR33 OS=Homo sapiens OX=9606 GN=WDR33 PE=1 SV=2 |
| Q9Y3Y2 | Chromatin target of PRMT1 protein | CHTOP | 12.095 | 10.5 | 2 | sp\|Q9Y3Y2\|CHTOP_HUMAN Chromatin target of PRMT1 protein OS=Homo sapiens OX=9606 GN=CHTOP PE=1 SV=2 |
| P46776 | 60S ribosomal protein L27a | RPL27A | 11.735 | 7.4 | 1 | sp\|P46776\|RL27A_HUMAN 60S ribosomal protein L27a OS=Homo sapiens OX=9606 GN=RPL27A PE=1 SV=2 |
| O76021 | Ribosomal L1 domain-containing protein 1 | RSL1D1 | 11.666 | 4.3 | 2 | sp\|O76021\|RL1D1_HUMAN Ribosomal L1 domain-containing protein 1 OS=Homo sapiens OX=9606 GN=RSL1D1 PE=1 SV=3 |
| P40937 | Replication factor C subunit 5 | RFC5 | 11.625 | 5.9 | 2 | sp\|P40937\|RFC5_HUMAN Replication factor C subunit 5 OS=Homo sapiens OX=9606 GN=RFC5 PE=1 SV=1 |
| P12236 | ADP/ATP translocase 3 | SLC25A6 | 11.277 | 22.5 | 2 | sp\|P12236\|ADT3_HUMAN ADP/ATP translocase 3 OS=Homo sapiens OX=9606 GN=SLC25A6 PE=1 SV=4 |
| Q8NEY8 | Periphilin-1 | PPHLN1 | 11.039 | 3.3 | 2 | sp\|Q8NEY8\|PPHLN_HUMAN Periphilin-1 OS=Homo sapiens OX=9606 GN=PPHLN1 PE=1 SV=2 |
| P40938 | Replication factor C subunit 3 | RFC3 | 10.979 | 6.5 | 2 | sp\|P40938\|RFC3_HUMAN Replication factor C subunit 3 OS=Homo sapiens OX=9606 GN=RFC3 PE=1 SV=2 |
| P52292 | Importin subunit alpha-1 | KPNA2 | 8.9922 | 2.8 | 1 | sp\|P52292\|IMA1_HUMAN Importin subunit alpha-1 OS=Homo sapiens OX=9606 GN=KPNA2 PE=1 SV=1 |
| P27348 | 14-3-3 protein theta | YWHAQ | 7.463 | 15.9 | 1 | sp\|P27348\|1433T_HUMAN 14-3-3 protein theta OS=Homo sapiens OX=9606 GN=YWHAQ PE=1 SV=1 |
| Q9Y2L9 | Leucine-rich repeat and calponin homology domain-containing protein 1 | LRCH1 | 7.3334 | 2.2 | 1 | sp\|Q9Y2L9\|LRCH1_HUMAN Leucine-rich repeat and calponin homology domain-containing protein 1 OS=Homo sapiens OX=9606 GN=LRCH1 PE=1 SV=3 |
| O14818; Q8TAA3 | Proteasome subunit alpha type-7; Proteasome subunit alpha-type 8 | PSMA7; PSMA8 | 7.1891 | 4.4 | 1 | sp\|O14818\|PSA7_HUMAN Proteasome subunit alpha type-7 OS=Homo sapiens OX=9606 GN=PSMA7 PE=1 SV=1;sp\|Q8TAA3\|PSMA8_HUMAN Proteasome subunit alpha-type 8 OS=Homo sapiens OX=9606 GN=PSMA8 PE=2 SV=3 |
| P09661 | U2 small nuclear ribonucleoprotein A | SNRPA1 | 7.0365 | 5.5 | 1 | sp\|P09661\|RU2A_HUMAN U2 small nuclear ribonucleoprotein A OS=Homo sapiens OX=9606 GN=SNRPA1 PE=1 SV=2 |
| P35249 | Replication factor C subunit 4 | RFC4 | 6.8938 | 3.6 | 1 | sp\|P35249\|RFC4_HUMAN Replication factor C subunit 4 OS=Homo sapiens OX=9606 GN=RFC4 PE=1 SV=2 |
| O95391 | Pre-mRNA-splicing factor SLU7 | SLU7 | 6.8715 | 2.9 | 1 | sp\|O95391\|SLU7_HUMAN Pre-mRNA-splicing factor SLU7 OS=Homo sapiens OX=9606 GN=SLU7 PE=1 SV=2 |
| Q9NUK0 | Muscleblind-like protein 3 | MBNL3 | 6.6757 | 2.5 | 1 | sp\|Q9NUK0\|MBNL3_HUMAN Muscleblind-like protein 3 OS=Homo sapiens OX=9606 GN=MBNL3 PE=1 SV=2 |
| Q9UKF6 | Cleavage and polyadenylation specificity factor subunit 3 | CPSF3 | 6.4624 | 1.5 | 1 | sp\|Q9UKF6\|CPSF3_HUMAN Cleavage and polyadenylation specificity factor subunit 3 OS=Homo sapiens OX=9606 GN=CPSF3 PE=1 SV=1 |
| Q14694 | Ubiquitin carboxyl-terminal hydrolase 10 | USP10 | 6.4113 | 1.9 | 1 | sp\|Q14694\|UBP10_HUMAN Ubiquitin carboxyl-terminal hydrolase 10 OS=Homo sapiens OX=9606 GN=USP10 PE=1 SV=2 |
| P61326; Q96A72 | Protein mago nashi homolog;Protein mago nashi homolog 2 | MAGOH; MAGOHB | 6.3168 | 7.5 | 1 | sp\|P61326\|MGN_HUMAN Protein mago nashi homolog OS=Homo sapiens OX=9606 GN=MAGOH PE=1 SV=1;sp\|Q96A72\|MGN2_HUMAN Protein mago nashi homolog 2 OS=Homo sapiens OX=9606 GN=MAGOHB PE=1 SV=1 |
| Q7Z2W4 | Zinc finger CCCH-type antiviral protein 1 | ZC3HAV1 | 6.2809 | 1.7 | 1 | sp\|Q7Z2W4\|ZCCHV_HUMAN Zinc finger CCCH-type antiviral protein 1 OS=Homo sapiens OX=9606 GN=ZC3HAV1 PE=1 SV=3 |
| P01877; P01876 | Immunoglobulin heavy constant alpha 2; Immunoglobulin heavy constant alpha 1 | IGHA2; IGHA1 | 6.2381 | 2.9 | 1 | sp\|P01877\|IGHA2_HUMAN Immunoglobulin heavy constant alpha 2 OS=Homo sapiens OX=9606 GN=IGHA2 PE=1 SV=4;sp\|P01876\|IGHA1_HUMAN Immunoglobulin heavy constant alpha 1 OS=Homo sapiens OX=9606 GN=IGHA1 PE=1 SV=2 |
| Q9UMS4 | Pre-mRNA-processing factor 19 | PRPF19 | 6.2351 | 1.6 | 1 | sp\|Q9UMS4\|PRP19_HUMAN Pre-mRNA-processing factor 19 OS=Homo sapiens OX=9606 GN=PRPF19 PE=1 SV=1 |
| Q15417 | Calponin-3 | CNN3 | 6.2215 | 4 | 1 | sp\|Q15417\|CNN3_HUMAN Calponin-3 OS=Homo sapiens OX=9606 GN=CNN3 PE=1 SV=1 |
| P55199 | RNA polymerase II elongation factor ELL | ELL | 6.1156 | 1.8 | 1 | sp\|P55199\|ELL_HUMAN RNA polymerase II elongation factor ELL OS=Homo sapiens OX=9606 GN=ELL PE=1 SV=1 |
| P35250 | Replication factor C subunit 2 | RFC2 | 6.08 | 2.3 | 1 | sp\|P35250\|RFC2_HUMAN Replication factor C subunit 2 OS=Homo sapiens OX=9606 GN=RFC2 PE=1 SV=3 |
| Q6ZVX7 | F-box only protein 50 | NCCRP1 | 6.0772 | 4 | 1 | sp\|Q6ZVX7\|FBX50_HUMAN F-box only protein 50 OS=Homo sapiens OX=9606 GN=NCCRP1 PE=1 SV=1 |
| Q13509 | Tubulin beta-3 chain | TUBB3 | -2 | 26 | 1 | sp\|Q13509\|TBB3_HUMAN Tubulin beta-3 chain OS=Homo sapiens OX=9606 GN=TUBB3 PE=1 SV=2 |
| O14936 | Peripheral plasma membrane protein CASK | CASK | -2 | 1 | 1 | sp\|O14936\|CSKP_HUMAN Peripheral plasma membrane protein CASK OS=Homo sapiens OX=9606 GN=CASK PE=1 SV=3 |
| O43663 | Protein regulator of cytokinesis 1 | PRC1 | -2 | 1.8 | 1 | sp\|O43663\|PRC1_HUMAN Protein regulator of cytokinesis 1 OS=Homo sapiens OX=9606 GN=PRC1 PE=1 SV=2 |
| Q9Y285 | Phenylalanine--tRNA ligase alpha subunit | FARSA | -2 | 2.2 | 1 | sp\|Q9Y285\|SYFA_HUMAN Phenylalanine--tRNA ligase alpha subunit OS=Homo sapiens OX=9606 GN=FARSA PE=1 SV=3 |
| Q86SR1 | Polypeptide N-acetylgalactosaminyltransferase 10 | GALNT10 | -2 | 1.5 | 1 | sp\|Q86SR1\|GLT10_HUMAN Polypeptide N-acetylgalactosaminyltransferase 10 OS=Homo sapiens OX=9606 GN=GALNT10 PE=1 SV=2 |
| P0DP72 | V-set and immunoglobulin domain-containing protein 10-like 2 | VSIG10L2 | -2 | 1.4 | 1 | sp\|P0DP72\|VSXL2_HUMAN V-set and immunoglobulin domain-containing protein 10-like 2 OS=Homo sapiens OX=9606 GN=VSIG10L2 PE=3 SV=1 |
| Q9NQW1 | Protein transport protein Sec31B | SEC31B | -2 | 0.7 | 1 | sp\|Q9NQW1\|SC31B_HUMAN Protein transport protein Sec31B OS=Homo sapiens OX=9606 GN=SEC31B PE=1 SV=1 |
